# Supplementary material for: Why are patients with acute traumatic brain injury not routinely assessed or treated for vestibular dysfunction in the UK? A qualitative study
Source: BMJ Open. 2023 Jan 2;13(1):e067967. doi: 10.1136/bmjopen-2022-067967 (PMC9809272; doi:10.1136/bmjopen-2022-067967)
Supplement: Supplementary data [file bmjopen-2022-067967supp001.pdf]

Supplementary Data

Supplementary File 1 - Topic guide

NHS Healthcare Professional Interview questions

Title: A qualitative study investigating the barriers and facilitators to screening of dizziness and imbalance in acute head injury patients.

Chief Investigator: Dr Barry Seemungal, Imperial College, Division of Brain Sciences, Charing Cross Hospital Campus, W6 8RF. Tel: 02033117042

Co-investigator: Rebecca Smith, Imperial College, Division of Brain Sciences, Charing Cross Hospital Campus, W6 8RF. Tel: 02033117042

Introduction

Thank you for agreeing to participate in this interview. The aim of the study is to explore healthcare professionals' knowledge and experience of assessing and treating dizziness in head injury patients. Your answers will be anonymous and will remain confidential. The interview will be audio recorded and I may take some notes whilst you are talking. Please keep in mind that you do not have to answer any questions you do not feel comfortable with, and we can stop the interview at any time.

Background questions:

1. Approximately how many patients with head injury with dizziness or imbalance do you see in a month? (i.e is dizziness a common symptom or sign after head injury?)

2. Can you talk me through the pathway of how head injury patients with dizziness or imbalance are managed?

3. Who is responsible for seeing these patients? (What do each of these team members do?)

4. What happens after they are assessed? (prompt: what is the treatment pathway? or onward referral procedure?)

We will now explore the pathway in detail using some more specific questions.

Memory, attention and decision process

5. Is assessing dizziness something you do routinely in head injury patients? (If not, why not?)

6. What factors might guide your decision to assess for dizziness in a head injured patient? (prompt: What clinical signs or symptoms are utilised?; formal assessments, on handover)

Knowledge

7. Which dizziness diagnoses are you familiar with post head injury? (Prompt: peripheral nerve injury; BPPV; migraine)

8. What sort of tests are you aware of that are useful in diagnosing dizziness? How are these test results used or interpreted? (prompt: are these standardized or validated tests?)

9. Are you aware of any treatment options for dizziness? (prompt: this might include onward referrals to other healthcare professionals)

10. Are there barriers to referring patients to appropriate services? (prompt: is there a wait for a specialist assessment?)

Skills

11. How confident are you in assessing and interpreting results of diagnostic tests for BPPV or other forms of dizziness?

12. What skills are required to treat dizziness conditions in head injury patients? (Prompt: BPPV)

Beliefs about capabilities

13. How would you rate your depth of understanding of dizziness? (prompt: How confident do you feel in managing dizziness after head injury?)

Social professional role and identity

14. Do you think conducting dizziness assessments is part of your role? If not, whose role does this fall into? (prompt: is assessing dizziness something you are trained to do?)

15. Do you think interpreting these results and providing treatment is an appropriate part of your role? Why / why not?

Beliefs about consequences

16. Are there any benefits of assessing and treating dizziness in these patients? (prompt: to yourself; patients, other clinicians; organisation).

17. Are there any consequences of 'not doing?' i.e. not assessing or treating? (prompt: is it a problem which resolves on its own; are there any consequences for the patient, clinicians, NHS, financial; long/short term) If no – why?

Motivation and goals

18. How important is it to assess and treat dizziness in head injury patients? (prompt: how high is the priority compared to other behaviours required to treat the patient?)

Environmental context and resources

19. Does the trauma ward situation or environment affect your management of dizzy head injury patients? (Prompt: i.e. are there time factors or competing tasks)

20. Are there clear communication channels if you are unsure of how to complete a test or interpret a result?

21. Are there any other person or environmental barriers or facilitators that might influence your screening behaviour? (prompt: guidelines/resources)

Social influences

22. Do other colleagues perform these tests? Does this influence your decision to complete / not complete these tests? (Prompt: AHPs; other medics)

23. How might the views of your colleagues affect you managing a dizzy head injured patient?

Emotion

24. Are there any situations in which you would be worried about managing dizziness in this population? (i.e medically/professionally/emotionally)

25. Are there any challenges in managing a head injury patient with dizziness?

Behavioral regulation

26. If you wanted to change your dizziness assessment behavior, how would you go about doing this? (prompt: what would facilitate you to do this?)

27. Is there any training that you or your team may require? (prompt: any procedures/guidelines/ways of working)

Lastly, is there any further you would like to add which we have not covered?

Thank you for participating in this interview.

Supplementary File 2 - Framework for analysis

1 Current practice

1.1 Identification and reporting of dizziness
1.2 Assessment and the assessment pathway
1.3 Treatment and referral pathway
1.4 Longer term effects of treating/not treating
1.5 Perceptions regarding role and responsibility relating to dizziness assessment and treatment
1.6 Views about specialist services

2 Dizziness and the clinical need

2.1 Factors relating to dizziness frequency, severity and longevity
2.2 Perceptions around the importance and clinical significance of dizziness
2.3 Impact of dizziness on patients
2.4 Impact of dizziness on service
2.5 Factors relating to early identification and intervention
2.6 Views about specific populations i.e. elderly or young

3 Current knowledge

3.1 Knowledge about BPPV as a condition
3.2 Knowledge and clinical reasoning relating to other causes of dizziness
3.3 Knowledge about diagnosis and treatment of BPPV
3.4 Healthcare professional’s ability to diagnose and treat dizziness conditions
3.5 Efficacy of BPPV treatment

4 Barriers to diagnosis and treatment competence

4.1 Views about the area and motivation/interest in the area
4.2 Factors related to training, skills or knowledge
4.3 Feasibility of diagnosis and treatment
4.4 Confidence
4.5 Role concerns
4.6 Prior surgical / medical clearance
4.7 Environmental factors
4.8 Lack of access to mentors or specialists
4.9 Tangible evidence of dizziness
4.10 Views about resources – staffing, workload, time

5 Changing behaviour and practice

5.1 Factors relating to role change
5.2 Facilitators to behaviour change
5.3 Benefits of practice change
5.4 Limitations or barriers to practice change
5.5 Training requirements
5.6 Strategies for changing or improving practice
5.7 Wider translatable aspects of behaviour change

Supplementary data 3 – Central Chart

	Profession	Gender	Speciality	Current role	View on clinical need	Assessment/Treatment knowledge & ability	Main barrier	Readiness for change
1 : AM12_1205	Medic, Junior Doctor	Male	Trauma	Adhoc, unspecific assessor, referrer	Low-medium	Partly theoretical, non functional	Resources & Clinical need	Reservations, not feasible to take on role
2 : AM13_1635	Medic, Registrar	Male	Trauma	Inactive, reliance on therapists	Low-medium	Theoretical, partly functional	Resources & Clinical need (view of the area)	Reservations, not feasible to take on role
3 : AM14_1530	Medic, Junior Doctor	Male	Trauma	Inactive	Low-medium	Partly theoretical, non functional	Current role & remit Resources & Clinical need	Reservations, not feasible to take on role (or just not engaged?)
4 : AM16_1445	Medic, Registrar	Female	Trauma	Inactive; reliance on therapists	Low-medium	Non theoretical, non functional	Resources & clinical need	Reservations, not feasible to take on role
5 : AM17_1700	Medic, Junior Doctor	Male	Trauma	Adhoc unspecific assessor, referrer	Low-medium	Non theoretical, non functional	Current role & remit	Reservations, not feasible to take on role
6 : AM21_1500	Medic, Junior doctor	Female	Trauma	Referrer	Low-medium	Non theoretical, non functional	Resources & clinical need	Minor reservations, not feasible to take on role
7 : AM271500	Medic, Registrar	Male	Trauma	Adhoc, unspecific assessor, referrer	Low-medium	Partly theoretical, non functional	Current role & remit	Reservations, not feasible to take on role
8 : AM281555	Medic, Junior Doctor	Female	Trauma	Adhoc, unspecific assessor, referrer	Low-medium	Partly theoretical, non functional	Resources & Clinical need	Reservations, not feasible to take on role
9 : AM8_1430	Medic, Consultant	Male	Trauma	Subjective/Objective identifier, unspecific assessor, referrer	Low-medium	Partly theoretical, non functional	Knowledge and skills	Reservations, not feasible to take on role
10 : AO1_1535	OT, Senior	Female	Trauma	Subjective/Objective identifier, unspecific assessor and referrer	High	Partly theoretical, non functional	Knowledge and skills	No reservations, keen to take on role
11 : AO15_1600	OT, Junior	Male	A&E	Routine identifier, unspecific assessor, referrer	Medium-high	Partly theoretical, non functional	Current role & remit	Reservations, not feasible to take on role
12 : AO18_1520	OT, Senior	Female	A&E	Routine identifier, unspecific assessor, referrer	Medium-high	Partly theoretical, non functional	Current role & remit	Reservations, not feasible to take on role
13 : AO19_1200	OT, Senior	Female	Neurology	Subjective identifier, unspecific assessor, referrer	Medium-high	Non theoretical, non functional	Current role & remit	Some reservations, uncertain about taking on the role
14 : AO20_1330	OT, Senior	Female	A&E	Subjective identifier, unspecific assessor, referrer	High	Theoretical, non functional	Current role & remit	Some reservations, uncertain about taking on role
15 : AO221447	OT, Senior	Female	Trauma	Routine identifier, screen, referrer	Medium-high	Theoretical, non functional	Current role & remit Confidence	Minor reservations, keen to take on role
16 : AO241030	OT, Senior	Female	Critical care	Subjective identifier, unspecific assessor, referrer	Low-medium	Theoretical, non functional	Current role & remit	Minor reservations, keen to take on role

17 : AO251130	OT, Senior	Male	Critical care	Subjective / objective identifier, unspecific assessor, referrer	Low-medium	Partly theoretical, non functional	Current role & remit	Minor reservations, keen to take on role
18 : AO4_1210	OT, Senior	Female	Trauma	Routine identifier, unspecific assessor, referrer	Medium-high	Partly theoretical, non functional	Current role & remit Knowledge and skills	Some reservations, uncertain about taking on role
19 : AO6_1005	OT, Senior	Female	Trauma	Routine identifier, unspecific assessor	High	Theoretical, non functional	Current role and remit Knowledge and skills	Minor reservations, keen to take on role.
20 : AO9_1230	OT, Junior	Female	Neurology	Routine identifier, unspecific assessor, referrer	Medium-high	Partly theoretical, non functional	Current role & remit	Minor reservations, keen to take on role
21 : AP10_2401	PT, Junior	Female	Neurology	Subjective identifier, unspecific assessor	Medium-high	Partly theoretical, non functional	Current role and remit Knowledge and skills	Some reservations, uncertain about taking on role
22 : AP11_1115	PT, Senior	Female	Trauma	Routine identifier, unspecific assessor, referrer	High	Theoretical, non functional	Low confidence	Minor reservations, keen to take on role
23 : AP2_1541	PT, Junior	Female	Neurology	Subjective identifier, unspecific assessor, referrer	Medium	Partly theoretical, non functional	Knowledge and skills	Minor reservations, keen to take on role
24 : AP231544	PT, Senior	Female	Neurology	Routine identifier, specific assessor, treat	Medium	Theoretical and partly functional	Confidence	Minor reservations, keen to take on role
25 : AP261400	PT, Senior	Female	Trauma	Subjective/objective identifier, unspecific assessor, referrer	Low-Medium	Theoretical, non functional	Current role & remit	Minor reservations, keen to take on role
26 : AP3_1030	PT, Junior	Male	Trauma	Subjective/objective identifier, referrer	High	Theoretical, partly functional	Confidence	Minor reservations, keen to take on role
27 : AP5_0840	PT, Junior	Female	Trauma	Subjective/objective identifier, unspecific assessor, referrer	Medium-high	Partly theoretical, non functional	Knowledge and skills	Minor reservations, keen to take on role
28 : AP7_1515	PT, Senior	Female	Trauma	Routine identifier, semi specific assessor, referrer	Medium-high	Partly theoretical, non functional	Knowledge and skills	Minor reservations, keen to take on role

Key: OT (Occupational Therapist); PT (Physiotherapist); Subjective/objective/routine identifier (referring to healthcare professional who is involved in routinely identifying patients with potential vestibular dysfunction through either subjective or objective means); specific/unspecific assessor (referring to a healthcare professional able to assess vestibular dysfunction using specific, semi specific or non specific assessment tools); referrer (referring to healthcare professional who refers onto specialists for further management); Theoretical (referring to healthcare professionals with theoretical knowledge of vestibular diagnoses, assessment and treatment); Functional /non functional (referring to healthcare professionals who utilise their vestibular knowledge and skills to assess and treat patients)
