# Supplementary material for: Why are patients with acute traumatic brain injury not routinely assessed or treated for vestibular dysfunction in the UK? A qualitative study
Source: BMJ Open. 2023 Jan 2;13(1):e067967. doi: 10.1136/bmjopen-2022-067967 (PMC9809272; doi:10.1136/bmjopen-2022-067967)
Supplement: Author's manuscript [file bmjopen-2022-067967.draft_revisions.pdf]

BMJ Open is committed to open peer review. As part of this commitment we make the peer review history of every article we publish publicly available.

When an article is published we post the peer reviewers' comments and the authors' responses online. We also post the versions of the paper that were used during peer review. These are the versions that the peer review comments apply to.

The versions of the paper that follow are the versions that were submitted during the peer review process. They are not the versions of record or the final published versions. They should not be cited or distributed as the published version of this manuscript.

BMJ Open is an open access journal and the full, final, typeset and author-corrected version of record of the manuscript is available on our site with no access controls, subscription charges or pay-per-view fees (http://bmjopen.bmj.com).

If you have any questions on BMJ Open's open peer review process please email info.bmjopen@bmj.com

BMJ Open

Why are patients with acute traumatic brain injury not routinely assessed or treated for vestibular dysfunction? A qualitative study

Journal:	*BMJ Open*
Manuscript ID	bmjopen-2022-067967
Article Type:	Original research
Date Submitted by the Author:	01-Sep-2022

Complete List of Authors:	Smith, Rebecca; Imperial College London, Brain and Vestibular Group, Centre for Vestibular Neurology<br>Burgess, Caroline; King's College London, School of Population Health & Environmental Sciences<br>Marsden, Jonathan; Plymouth University, School of Health Professions<br>Seemungal, Barry; Imperial College London, Brain and Vestibular Group, Centre for Vestibular Neurology,

Keywords:	Neurotology < OTOLARYNGOLOGY, TRAUMA MANAGEMENT, REHABILITATION MEDICINE, QUALITATIVE RESEARCH

SCHOLARONE™
Manuscripts

I, the Submitting Author has the right to grant and does grant on behalf of all authors of the Work (as defined in the below author licence), an exclusive licence and/or a non-exclusive licence for contributions from authors who are: i) UK Crown employees; ii) where BMJ has agreed a CC-BY licence shall apply, and/or iii) in accordance with the terms applicable for US Federal Government officers or employees acting as part of their official duties; on a worldwide, perpetual, irrevocable, royalty-free basis to BMJ Publishing Group Ltd ("BMJ") its licensees and where the relevant Journal is co-owned by BMJ to the co-owners of the Journal, to publish the Work in this journal and any other BMJ products and to exploit all rights, as set out in our licence.

The Submitting Author accepts and understands that any supply made under these terms is made by BMJ to the Submitting Author unless you are acting as an employee on behalf of your employer or a postgraduate student of an affiliated institution which is paying any applicable article publishing charge ("APC") for Open Access articles. Where the Submitting Author wishes to make the Work available on an Open Access basis (and intends to pay the relevant APC), the terms of reuse of such Open Access shall be governed by a Creative Commons licence – details of these licences and which Creative Commons licence will apply to this Work are set out in our licence referred to above.

Other than as permitted in any relevant BMJ Author's Self Archiving Policies, I confirm this Work has not been accepted for publication elsewhere, is not being considered for publication elsewhere and does not duplicate material already published. I confirm all authors consent to publication of this Work and authorise the granting of this licence.

For peer review only

Why are patients with acute traumatic brain injury not routinely assessed or treated for vestibular dysfunction? A qualitative study

Rebecca M Smith[1†], Caroline Burgess[2], Jonathan Marsden[3], Barry M Seemungal[1†]

1. Brain & Vestibular Group, Centre for Vestibular Neurology, Imperial College London
2. School of Population Health & Environmental Sciences, King's College London
3. School of Health Professions, University of Plymouth

†Corresponding authors

10L14 Laboratory Block

Charing Cross Campus

London

W6 8RP

Email: Rebecca.smith@imperial.ac.uk; b.seemungal@imperial.ac.uk

For peer review only

ABSTRACT

Objectives

Vestibular dysfunction is common in patients with acute traumatic brain injury (aTBI). Persisting vestibular symptoms (i.e. dizziness and imbalance) are linked to poor physical, psychological, and socioeconomic outcomes. However, routine management of vestibular dysfunction in aTBI is not always standard practice. We aimed to identify and explore any person-related barriers or facilitators to managing vestibular dysfunction in aTBI.

Design

A qualitative approach was used. Data was collected using face to face, semi-structured interviews and analysed using the Framework approach.

Setting

Two Major Trauma Centres in London, UK.

Participants

28 healthcare professionals participated: 11 occupational therapists, 8 physiotherapists and 9 surgical/trauma doctors.

Results

Vestibular assessment and treatment was not routinely undertaken by trauma ward staff. Uncertainty regarding responsibility for vestibular management on the trauma ward was perceived to lead to gaps in patient care. Interestingly, the term dizziness was sometimes

For peer review only

perceived as an invisible and vague phenomenon, leading to difficulties identifying or ‘proving’ dizziness and a tendency for making non-specific diagnoses. Barriers to routine assessment and treatment included limited knowledge and skills, a lack of local or national guidelines, insufficient training, and concerns regarding the practical aspects of managing vestibular dysfunction. Of current trauma ward staff, therapists were identified as appropriate healthcare professionals to adopt new behaviours regarding management of a common form of vestibular dysfunction (benign paroxysmal positional vertigo). Strategies to support this behaviour change include heightened clarity around role, implementation of local or national guidelines, improved access to training, and multidisciplinary support from experts in vestibular dysfunction.

Conclusions

This study has highlighted role and knowledge barriers exist to MDT management of vestibular dysfunction in aTBI. Trauma ward therapists were identified as the most appropriate healthcare professionals to adopt new behaviours. Several strategies are proposed to facilitate such behaviour change.

For peer review only

Article Summary

Strengths and limitations of the study

- This multi-centre qualitative study is the first to explore barriers and facilitators to managing vestibular dysfunction in aTBI in a range of healthcare professionals.
- Data were analysed using a systematic, transparent approach, framework analysis, to heighten rigour and trustworthiness within the results.
- Whilst the study size was modest, healthcare professionals were purposefully sampled to encompass a range of professional backgrounds and experience.
- Although patients were recruited from two sites, this represents half of the major trauma centres in London, UK, supporting the transferability of findings to other urban major trauma centres in the UK.
- This study investigates healthcare professionals' barriers and facilitators to managing vestibular dysfunction in aTBI, future work could usefully explore patients' and carers' experiences.

Word count: 4985

For peer review only

INTRODUCTION

Vestibular dysfunction in TBI – linked to injury of peripheral (i.e. inner ear and nerve) or central (i.e. brain) vestibular structures can result in dizziness or imbalance [1] and is very common, affecting up to 80% of ambulant moderate-to-severe aTBI patients [2,3]. Vestibular dysfunction in aTBI may be caused by a range of diagnoses including benign paroxysmal positional vertigo (BPPV), centrally mediated gait ataxia (typically a 'vestibular ataxia'), migraine phenotype headache and acute peripheral unilateral vestibular loss [4]. Patients typically present with multiple vestibular diagnoses [4,5], increasing the complexity of assessment and treatment and elevating the risk of missed diagnoses.

Early management of vestibular dysfunction following TBI appears to be important. Delays to or inaccurate diagnosis and treatment may adversely impact patients' physical and psychosocial outcomes and quality of life [6–8], whilst persisting vestibular symptoms have been found to delay return to work [9]. Vestibular dysfunction, by its link to falls [10–12] (which affect half of TBI survivors [13]), results in significant physical, psychological and healthcare costs [14–16]. Evidence also points to the direct impact of vestibular dysfunction on mental wellbeing, with studies reporting links between the vestibular system and brain areas involved in emotional and cognitive processing [17–19]. Despite the need for early intervention, routine vestibular assessment and treatment during aTBI does not appear to be commonplace. Previous studies noted long delays to diagnosis and treatment [8], and large discrepancies in assessment and treatment practices between trauma centres [20]. Potential explanations for a lack of routine vestibular management in aTBI include: (i) limited ward based vestibular expertise (ii) absence of recommendations for vestibular assessment or treatment in national early management of head injury guidelines [21] and (iii) a newly described but common clinical phenomenon called vestibular agnosia (blunting of vestibular perception in patients with moderate-severe aTBI) [22].

For peer review only

The lack of vestibular expertise on major trauma wards is perhaps not surprising since the complexity of vestibular dysfunction has only recently been elucidated [4,5,22], whilst vestibular neurologists / specialists do not appear in the current list of recommended trauma ward based healthcare professionals [23]. Whether the latter is a consequence of or contributing factor to national guidelines not stipulating the need of routine vestibular assessment or management is unclear, however the two factors may well be interdependent. Thus, major trauma wards are traditionally staffed by surgical specialities who are expert at acute life-saving interventions but less so for managing the neurological complications of TBI (dizziness, imbalance, and cognitive sequelae). Perhaps confusing for healthcare professionals is the finding that vestibular dysfunction in aTBI, with its attendant increased risk of falls, can be 'silent' because of disrupted vestibular perception causing a vestibular agnosia (linked to a seven-fold reduction in recognition of common diagnoses such as BPPV by ward staff) [22]. Traditional teaching for healthcare professionals is to perform a focussed examination based upon the history. However, in aTBI, the more significant the brain injury the higher the underlying vestibular burden, but the less likely the patient is to report symptoms. For example, BPPV is twice as common in aTBI with skull fracture than in patients without skull fracture; i.e. 33% vs. 66% [24]. The combination of a very high vestibular burden in aTBI, its silent nature, and the standard approach to perform symptom-specific examination results in many patients being discharged home without any vestibular diagnoses, let alone specific treatment.

In summary, although early vestibular assessment and treatment in all aTBI patients is warranted, it is not routinely implemented. To consider any change in policy or practice, it is imperative to understand the views of those routinely delivering the service or those who are likely to be affected by it beforehand [25]. Accordingly, we aimed to explore person related barriers or facilitators associated with screening, assessment, and treatment of vestibular dysfunction in aTBI patients.

For peer review only

MATERIALS AND METHODS

Study Design

A qualitative methodology, the Framework approach, was used to gather experiential data [26]. Originally this methodology was developed for large scale policy research, but has been utilised more widely in health research [27] and pertinently, in studies exploring barriers to diagnostic and treatment implementation [28–31].

Sampling, Sample Size and Participants

Clinicians were invited by email to participate if (i) they routinely worked on a trauma, emergency, outlying, or rehabilitation ward receiving patients directly from acute trauma wards, and (ii) had a role in routine assessment and treatment of aTBI patients. Purposive sampling was used to obtain a sample of 28 healthcare professionals with differing levels of experience. Guidance from previous studies using Framework analysis and discussions regarding data saturation were used to determine an appropriate sample size [32,33]. This sampling method is in line with similar studies [34,35].

Data collection and setting

Semi-structured, individual, face-to-face interviews were conducted by the same researcher (RS), using a topic guide (Supplementary file 1) to gather in-depth data [36–38]. Interviews were audio-recorded and transcribed verbatim. Written consent was obtained from all participants.

The theoretical domains framework (TDF) was utilised to inform the topic guide. The TDF was used to (i) allow a greater understanding of factors influencing clinical behaviour, (ii) determine possible strategies to change behaviour, and (iii) clarify how such strategies might be best executed [39]. The TDF was developed to identify psychological and organisational theory relevant to health practitioner behaviour change; culminating in 12 domains covering

For peer review only

factors such as knowledge, skills and social and professional roles [40]. The TDF may therefore provide a theoretical model for any subsequent behaviour change intervention, and further, may enable successful implementation of that intervention. The topic guide included questions on how symptoms of vestibular dysfunction i.e. dizziness and imbalance were managed on the trauma ward. Participants were not asked to define dizziness or imbalance as this did not form part of the primary research question.

Data analysis

Data were analysed utilising the Framework approach; a series of interconnected stages enabling the researcher to move back and forth across the data until a coherent account emerges. During analysis the data is charted, and sorted according to key themes [41]. Two researchers reviewed and refined the framework (Supplementary file 2) which underwent several iterations before it encompassed the whole data set. Using NVivo (v.12), the data were charted, whereby indexed data were summarised for each participant. After charting ten transcripts, data were discussed amongst the research team. Some subthemes were noted to be redundant or hold significant overlap and were removed or renamed. Following refinement of the framework, the final transcripts were charted.

Mindmaps (MindView v.7) were created for each of the five themes, providing a visual representation of the whole data set. Notes were made on connections, patterns and areas of convergence and divergence between participants, subthemes, or themes. Finally, a central chart (Supplementary file 3) was created encompassing all respondents across both sites. This was used to explore patterns across themes and participant groups.

Patient and public involvement

Patients were involved in aiding identification and prioritisation of the research question and study design. More specifically, during an information session, patients from a local TBI

For peer review only

association reported poor experiences of vestibular dysfunction management in aTBI, whilst healthcare professionals were perceived to poorly understand symptoms of vestibular dysfunction. Such feedback formed the basis of the present study.

RESULTS

Results are reported in accordance with Consolidated criteria for reporting qualitative research (COREQ) guidelines where possible [42]. Thirty-five healthcare professionals across two Major Trauma Centres were invited to take part: of those, 28 participated. Table 1 illustrates their demographics.

Healthcare Professional	Number interviewed	Female	Speciality (number)
Junior Therapist (Band 5-6)	7	5	Physiotherapist (4)<br>Occupational Therapist (3)
Senior Therapist (Band 7-8)	12	10	Physiotherapist (4)<br>Occupational Therapist (6)
Junior doctor (FY1-2)	5	2	Surgery (3)<br>Trauma (2)
Senior doctor (Registrar-Consultant)	4	1	Neurosurgery (2)<br>Trauma (2)

Table 1. Demographics of study participants.

Five main themes relating to healthcare professionals' experiences of managing vestibular dysfunction are outlined; (i) Current practice – who is responsible for screening, assessing, and treating vestibular dysfunction? (ii) The invisibility of dizziness: how clinically important is it? (iii) How confident are healthcare professionals in their knowledge and skills to assess and treat vestibular dysfunction? (iv) What are the practical barriers to assessment and treatment? and (v) Who and what is required for behaviour change? These five main factors

For peer review only

were noted to be connected by an overarching characteristic: Healthcare professionals' role (Figure 1).

Figure 1 here

Figure 1. Five main themes and their relation to the overarching concept of 'role'.

Current practice – who is responsible for screening, assessment, and treatment of vestibular dysfunction?

Trauma and surgical doctors across both sites felt a theoretical sense of oversight for all trauma related deficits, however there was uncertainty regarding responsibility for day-to-day management of vestibular dysfunction. Doctors appeared less likely to ask about dizziness or assess vestibular dysfunction routinely. However, one divergent case, a consultant neurosurgeon, reported screening patients more frequently although this was guided by the presence of skull fractures on a scan, rather than a routine question.

> *'No, we absolutely don't assess it routinely… it's not something that we directly asked questions about, so we often missed it'*
>
> *[A13, Registrar, surgery]*

Therapists (physiotherapists and occupational therapists) also expressed uncertainty about responsibility for managing vestibular dysfunction, although in practice they tended to identify patients and coordinate referrals. Therapists attributed taking on these roles to spending more time with patients and being the first healthcare professional to mobilise or complete functional tasks with patients (these activities often provoked dizziness). Large

For peer review only

variability was noted in how therapists identified patients; some routinely asked about dizziness or imbalance, whereas others relied on patient report or manifestations of vestibular dysfunction in patients' balance, gait, or body language. Following identification, therapists referred patients to specialists. No therapists at either site reported completing specific assessments or treatments independently. There was a sense amongst occupational therapists in particular, that managing vestibular dysfunction was outside of their remit and instead embedded in a physiotherapist's role (due to their existing involvement in balance assessment).

> *'I think when you're an OT, probably the expectations were that you're not the one doing that. I would have no problem to do it if I felt that I was trained to do it. I think it's just never been something I've ever been encouraged to look into because, generally, the physios come along and do it…I think it might be a cultural thing within therapy'*
>
> *[A6, Occupational therapist, Senior Specialist]*

Respondents reported variability concerning when and to whom patients were referred. At both sites, specialists were visiting teams and therefore not regularly ward or sometimes even site based, leading to variation in time-to-assessment and occasionally patients being discharged prior to assessment. Interestingly, although specialist input was universally noted to be positive, respondents also viewed their presence as a barrier to improving their vestibular knowledge and skills. Uncertainty was also evident in which patients were eligible for follow up post-discharge. Therapists appeared to devolve responsibility to patients, thought to be a sub-optimal process due to concerns patients would 'fall through the net'. The uncertainty around responsibility and the variability noted in treatment pathways was thought to have a negative impact on care during and following admission.

For peer review only - http://bmjopen.bmj.com/site/about/guidelines.xhtml

'I don't know about a clear pathway, there's not really a protocol I should say, it's bit more adhoc…The service is not really equitable for everybody, and also probably people are lost to follow up and have poor outcomes in the future'

[A5, Physiotherapist, Senior]

The invisibility of dizziness: How clinically important is it?

There were marked differences between doctors and therapists regarding their perception of the clinical importance of dizziness, seemingly related to expectations of their role. Doctors perceived dizziness as a short-lived symptom and where it did persist, it could be managed in outpatient rather than in-patient settings.

'Not a massive priority…I think the feeling is that if someone has a head injury, they probably have a bit of, they could well have some dizziness, but it may not you know, it'd probably just be transient'

[A27, Registrar, trauma]

Doctors' views on the importance of dizziness appeared to be related to their clinical priorities of immediate life preservation or signs of acute deterioration. Once patients were more stable, they could 'take their foot off the pedal' which seemingly manifested in how frequently they asked about vestibular symptoms. Doctors noted the impact of dizziness on patients' confidence and balance, but generally were more ambivalent about it causing direct harm. Notably there was the feeling that it could delay discharge.

'These patients are not so bad that they have to be seen within 24 hours, even if you delayed it [treatment] by two or three days it's not a big issue. It increases their hospital stay but it doesn't cause any harm to them, so it's not a big problem'

For peer review only

[A8, Consultant, neurosurgeon]

Interestingly, ‘subtle’ or ‘invisible’ were words used to describe dizziness, attributed to ward round assessments being conducted whilst patients were lying still when signs of vestibular dysfunction were not always apparent (to a non-expert) and/or dizziness was not reported. Further, respondents noted the subtlety, subjective and positional nature of dizziness was not only a barrier to identification (and therefore accurate assessment and treatment), but also in ‘proving’ patients were dizzy, which was further seen to limit its clinical importance. Respondents did not mention the potential for objective measurement of vestibular dysfunction and hence were likely unaware of the capability for definitive diagnosis via laboratory testing.

‘It’s not seen on a scan and so often it’s the importance of it is hard to emphasise to the wider sort of medical community so whereas if it’s a blood result you can show it and I think the impact is probably underestimated. As physiotherapists we probably know the impact of it but generally, I’m not convinced the wider medical community recognises the repercussions.’

[A3, Physiotherapist, Senior]

Therapists viewed dizziness as a higher priority; increasing risk of falls, impacting cognition, attention, confidence, independence, and emotional and social wellbeing. Additionally, dizziness was noted to impede progress with recovery, resulting in an increased length of stay, heightened demand on ward therapy staff, and more support at home.

‘If they’re feeling dizzy, they spend longer periods in bed and they’re up and walking around less, which then obviously has a lot of other secondary complications in terms of prolonged bed rest and not moving around and decreased appetite or decreased oral intake, just because they’re struggling to get up. And then I think it can increase their length of stay”

[A11, Physiotherapist, Senior Specialist]

For peer review only

How confident are healthcare professionals in their knowledge and skills to assess and treat vestibular dysfunction?

Across all respondents, there was some theoretical knowledge but limited ability or confidence with practical vestibular assessment and treatment skills. Therapists denied knowledge or use of vestibular assessment tools, whilst there was wider awareness and use of 'balance measures' and assessment for postural hypotension. Low confidence was noted in undertaking eye movement examinations, and interpretation of findings was felt to be out of remit.

> *'I don't routinely do an actual dizziness assessment. I'd maybe look into balance and see whether they've got poor balance which might be linking everything in... I don't have a specific assessment to do'*
>
> *[A10, Physiotherapist, Junior]*

Respondents exhibited some theoretical knowledge of how to undertake clinical bedside diagnostic and treatment manoeuvres for the most common cause of peripheral vestibular dysfunction (BPPV), but little or no confidence in practical skills. Where there was practical experience, this was limited to physiotherapists who were not routinely treating patients due to (i) low confidence secondary to limited patient exposure and insufficient training and mentorship, (ii) a reliance on visiting specialists, or (iii) the practicality of undertaking treatment in aTBI patients. Across both sites therapists had little confidence in other therapy or medical staff to manage BPPV. Therapists noted specialists were effective at treating BPPV, although there was divergence regarding dosing and optimum time to treat.

> *'I don't think any of us up here feel confident to do it…I also think because the [trauma] doctors are rotational and don't necessarily have in-depth knowledge, they*

For peer review only

won't be confident to prescribe or treat. They definitely don't know how to do the manoeuvres'

[A5, Physiotherapist, Senior]

Similarly, trauma or surgical doctors did not report use of specific vestibular assessments. There was more confidence in completing general eye movement examinations, although interpretation of findings and discerning peripheral (i.e. inner ear) versus central (i.e. brain) patterns was felt to be complex. Some theoretical knowledge of the BPPV diagnostic test was apparent, but there were lower levels of confidence in their (or their therapy or medical colleagues) ability to practically undertake bedside tests or treatment. Non-specific treatments such as medication to supress vertigo were most frequently reported as first-line treatment.

'I don't think anyone in my team including myself would confidently say we can deal with it…I don't think the therapists would go to the extent of actually doing specific manoeuvres, the Hallpike manoeuvre'

[A8, Consultant, neurosurgeon]

What are the practical barriers to assessment and treatment of vestibular dysfunction?

Participants perceived training and knowledge as the most fundamental barrier to managing vestibular dysfunction. Secondary barriers were both intrinsic (motivation and confidence) and extrinsic (time and the feasibility of completing diagnostic and treatment procedures). Whilst both physiotherapists and occupational therapists felt their previous vestibular training

For peer review only

did not enable them to assess and treat independently, occupational therapists perceived their training was not comparable to that of physiotherapists. Although neither physiotherapists nor occupational therapists felt managing vestibular dysfunction was an expectation of their role, occupational therapists felt concerned that taking an interest would cross professional boundaries. Dizziness and imbalance were still perceived to be relevant to their role, however.

> *'I'm not sure whether we would be um sort of stepping in some area that is not supposed to be ours however in terms of occupational therapy, it's something that affects function so I think it's relevant…we should be more aware of how to treat'*
>
> *[A4, Occupational therapist, Junior]*

For those receiving more training, limited or adhoc exposure to patients and hence reduced application of practical skills, coupled with the rotational nature of training seemingly affected confidence, knowledge, and skill consolidation.

> *'I haven't actually been taught how to do it [assessment and treatment of BPPV], I've just been shown it, or told the basis behind it, rather than actually being taught to carry it out…I think rotating out of it all the time and just not, never really having the chance to consolidate skills… again it's not really something that we're expected to manage'*
>
> *[A11, Physiotherapist, Senior Specialist]*

Doctors recalled some undergraduate teaching but felt this was not revisited during clinical training, perhaps associated with their focus on managing acute aspects of TBI. Further,

For peer review only

there was a perception amongst doctors at both sites of dizziness as an ill-defined symptom, without discrete cause or diagnosis and without specific treatment. This view of dizziness as ‘unfixable’ seemed particularly important for neurosurgeons who were accustomed to being able to ‘fix’ symptoms. Interestingly, this perception was not noted amongst therapists.

> *‘There is this concept of post-concussion dizziness where it’s really non-specific and the idea is it’s not really treatable, it’s not a specific condition. And so if it’s not treatable and particularly as surgeons our mindset is to only think about things you can fix…I think internationally that post-concussion dizziness isn’t discrete diseases, it’s just a fluffy phenomenon that occurs as a sequelae of head injury’*
>
> *[A13, Registrar, surgery]*

Time constraints were particularly evident amongst doctors and therapists working in emergency areas, seemingly due to competing priorities. Contrastingly, trauma therapists highlighted two secondary barriers (i) paucity of role models (ii) feasibility of identifying vestibular dysfunction and performing assessments and treatments in aTBI patients due to spine and limb fractures, pain, consciousness, communication, cognition, and insight.

Who and what is required for behaviour change?

When asked about incorporating managing vestibular dysfunction into their role, trauma therapists displayed the most enthusiasm about behaviour change. This was corroborated by doctors, who perceived therapists to have adequate time and to see patients at an appropriate point in their recovery. Trauma therapists were specific about solely managing BPPV, rather than other causes of vestibular dysfunction.

For peer review only

'If I had the right training, I'd be very happy to go and treat, considering that it's [BPPV treatment] almost 100% effective and its quicker turnaround, absolutely. I don't think, why would we need to waste more resources and time really? Because what am I just screening in order to get someone else to come and fix the problem? So, it might be better if we were trained, I think it would be in our scope'

[A25, Occupational therapist, Senior Specialist]

Reservations to behaviour change included (i) concerns regarding remit (occupational therapists) and (ii) concerns around staff capacity and transferability of skills (physiotherapists). Facilitators involved changes to local or national guidelines and accessible information for healthcare professionals' and patients to help consolidate awareness and knowledge.

"Some sort of policy or practice because I don't think it's enough just to do some training. Because I think people go for these trainings, then just don't do it. I think has to maybe come from something bigger, like a change in policy…. or something locally like a policy on head injury management or vestibular management"

[A7, Physiotherapist, Team Lead]

Role-related facilitators included setting clear expectations, regular patient exposure, vestibular neurology team support for complex cases, training and mentorship, and endorsement from line managers. Heighted theoretical and practical training and training a range of therapists to ensure sufficient capacity and maintenance of skills was felt necessary. A theoretical and practical checklist was thought to improve confidence and

For peer review only

ensure consistency. Potential benefits to behaviour change included more timely assessment and treatment, shortened hospital stay, improved progress with therapy and fewer patients with BPPV being missed.

> *"I think it would only benefit the patient because actually earlier on we'd be focusing on all aspects and picking it up better... And I think as well if you can settle someone's dizziness earlier on, they'll engage better with the therapy along the way because actually if they're always dizzy and we're struggling with that for a while and we're waiting for that assessment and then the treatment, you know you want the patient to build confidence"*
>
> *[A26, Physiotherapist, Senior Specialist]*

DISCUSSION

Summary of findings

This study provides new insights into the impact of healthcare professional's perceived role on vestibular assessment and treatment behaviours in aTBI. Our findings suggest management of vestibular dysfunction may be affected by (i) uncertainty within healthcare professionals' role and assessment and treatment pathways, (ii) self-reported lack of confidence in practical skills, (iii) lack of training, access to a multi-disciplinary team for complex cases, and mentorship and (iv) a perception of dizziness as an invisible and un-fixable entity. Optimism was expressed however, towards the possibility of behaviour and role change to improve the care pathway.

For peer review only

The role-related uncertainty displayed by healthcare professionals may be linked to lack of detail in existing guidelines in how, when and by whom post-traumatic vestibular dysfunction should be managed [21,23]. Previous research notes sufficient clarity surrounding role is significant in relation to completing and focusing on important tasks [43], whilst implementation of guidelines in other areas of aTBI management noted improved practice and cost savings [44]. Thus, heightened clarity around clinicians' roles and formation of a care pathway or guidelines may be useful in improving care for aTBI patients with vestibular dysfunction.

Insufficient training and a scarcity of role models were also perceived to be barriers to routine care. The degree to which vestibular management is included in undergraduate medical or therapy training is unclear, however published surveys note training is variable and appears to be undertaken at postgraduate level [45,46]. To date there are no formal qualifications for therapists involved in managing general vestibular dysfunction although draft proposals are in process for physiotherapists [47]. Such subspeciality training could involve integration with a larger infrastructure providing access to expert review and audit of complex cases, training, research opportunities and mentorship. The lack of available role models noted by our participants is also noteworthy given documented benefits of clinical behaviour, identity and career development [48,49]. Indeed, the absence of role models may have contributed to the ambiguity around responsibility for patient care and thus would be important to address for behaviour change.

Our participants described the term dizziness as an invisible or subtle phenomenon. Previous research corroborates this, noting (i) healthcare professionals perceive dizziness to be a vague symptom [50]; (ii) patients themselves exhibit inconsistencies in subjective reporting of dizziness [51] and (iii) when patients were asked about their perceptions of living

For peer review only

with dizziness, ‘invisible’ was commonplace [52,53]. Importantly, these latter studies noted patients with chronic dizziness associated invisibility with a lack of self-validation and validation from healthcare professionals, thus giving weight to the rationale for early diagnosis and treatment in aTBI patients. Dizziness was described as ‘subtle’ by participants in our study, despite objective signs of vestibular dysfunction being elicitable (albeit by experts) in the majority of aTBI patients [4,24]. Given many patients complain of few or no vestibular symptoms (due to vestibular agnosia) [22], it is perhaps not surprising that aTBI patients’ perceived dizziness is indeed, subtle. It follows that trauma and surgical doctors view of dizziness as a non-specific entity, without need for further diagnosis or specific treatment is understandable, although incorrect given the latest research [4,22,24]. Indeed, the general term ‘post-traumatic dizziness’ has historically been used in literature and practice rather than specific diagnoses, although recent work highlights the need to diagnose discrete conditions [4,5,54]. A specific diagnosis is important for accurate treatment and for self-validation [53,55], which in turn can influence attitudes and beliefs [56]. Further high-quality treatment studies may encourage the use of specific vestibular diagnoses amongst healthcare professionals, thus providing patients with accurate treatment and validation of their symptoms.

Figure 2 here

Figure 2. Diagram noting the themes contributing to the ‘invisibility’ of vestibular dysfunction

Surgical and trauma ward doctors assumed oversight for trauma patient care, however due to their focus on life-threatening complications of aTBI and the specialist nature of managing vestibular dysfunction, they tended to feel managing dizziness and imbalance were better suited to visiting specialists. Therefore, it is not perhaps practical to expect surgical or trauma doctors to acquire the necessary competency to manage vestibular dysfunction, and

For peer review only

thus new models of care should incorporate clinicians who either already were sufficiently trained or had capacity to be trained and mentored. From our sample trauma therapists (physiotherapists and occupational therapists) felt most able to adopt new assessment and treatment behaviours, limited to managing BPPV rather than all causes of post-traumatic vestibular dysfunction. This is perhaps not surprising as vestibular presentations may interact with other complex post-traumatic neurological conditions such as epilepsy, where some treatments may worsen dizziness and imbalance and delay discharge [57], or vestibular migraine whose commonest acute manifestation is gait ataxia [58]. An optimal scenario may thus involve a team; therapists trained, supported, and mentored by a clinician, such as a vestibular neurologist, who is also capable of managing complex vestibular presentations, allowing the provision of a comprehensive aTBI neurological service. National and/or local guidelines delineating which cases would benefit from this service would improve management within the aTBI cohort. Useful strategies may additionally include formation of a therapy led group from UK major trauma centres with the aim of developing peer support and sharing research, experiences, and training. Such strategies have been found to be effective in other areas [59]. There is precedence for physiotherapists taking on vestibular roles, however few occupational therapists appear to practice in this area in the UK [20]. This trend is not limited to TBI, as a predominance of physiotherapists work with vestibular patients in other albeit mainly outpatient areas [46,60]. However, physiotherapists and occupational therapists role can and do overlap, as noted by studies of in-patient stroke rehabilitation where such overlap was found to benefit patient care [61]. In-patient, multidisciplinary settings such as trauma, could therefore afford an environment where overlap of roles may provide additional staff capacity and support (highlighted to be a concern by respondents in our study). Acknowledging any reservations and working with all healthcare professionals involved would be key to improving future care pathways. Whether therapist led changes in behaviour would improve patient and service pathways remains unclear, however there is evidence in other settings such changes can reduce referrals and hospital visits [62].

For peer review only

To implement a change in behaviour it is necessary to ensure the intervention to change the identified behaviour is well designed [63,64], preferably using a framework to ensure relevant factors are considered [63]. Using the Behaviour change wheel [65], suggestions for appropriate intervention functions and associated policies are shown in Table 2.

Essential condition	Intervention	Policies	BPPV specific strategy
Capability	Education	Communication	Providing examples of therapists managing BPPV in other clinical areas
		Guidelines	National or local guidelines recommending vestibular assessment and treatment in acute TBI
	Training	Communication	Regular teaching on theoretical and practical assessment and treatment techniques for BPPV
Opportunity	Enablement	Service provision	Ensuring therapy teams have sufficient clinical capacity, role models and managerial support
			Medical (Consultant level) support for complex cases
		Regulation	Senior therapists being seen to use skills and train junior therapists
			Making relevant teams and individuals aware of a change in practice and a change in role
	Modelling	Communication	Clear expectations of what the role would involve
		Regulation/Guidelines	Embedding a pathway of care for patients with vestibular diagnoses in TBI into acute services
			Data showing which patients would benefit from assessment and treatment
Motivation	Persuasion	Communication	Using data to show patient and service level benefits of assessing and treating patients early

Table 2. Table showing possible behaviour change interventions for therapists

Limitations

Although this was a multi-centre study, the two participating sites were from a similar locality, and thus the generalisability of our findings may be limited. Whilst the participants sampled had a range of experience, we recruited only therapists and trauma and surgical doctors. Specialist brain injury nurses could have also been sampled, however previous literature notes therapists and doctors are most commonly involved in managing ward patients with vestibular dysfunction [46]. Notably, the involvement of neurologists with vestibular expertise in assessing aTBI – as occurs at our Trust - is uncommon in the UK. Finally, we did not ask participants to define dizziness at the outset of interviews. This may have resulted in slight subjective differences in participants' meanings of the term dizziness and should be considered when interpreting findings.

CONCLUSIONS

This multi-centre qualitative study highlights the barriers and facilitators to providing timely and accurate care for aTBI patients with vestibular dysfunction. A range of role, knowledge, and practical barriers to managing vestibular dysfunction in aTBI were noted. Within our sample, trauma therapists appeared most suited to incorporate new assessment and treatment behaviours for BPPV into routine practice, supported by an appropriately trained MDT. Theory based strategies for implementing interventions to change behaviour are proposed. Further work is required to establish whether such changes in behaviour would result in patient and/or service level improvements.

Author Contributions

All authors contributed to this work. RS designed/conceptualised the study/collected data/analysed data and drafted the manuscript. RS holds a MRes and is an NIHR Doctoral

For peer review only

Fellow and a physiotherapist by background. CB designed/conceptualised the study/analysed data and revised the manuscript. JM designed/conceptualised the study and revised the manuscript. BMS designed/conceptualised the study/revised the manuscript and supervised the study.

Competing interests

RS, CB, and JB declare no competing interests. BMS is an ABN traumatic brain injury advisory committee member, a NICE guidelines review committee for head trauma and Editor in Chief for Journal of Concussion

Funding statement

Rebecca Smith is funded by the National Institute of Health Research [ICA-CDRF-2017-03-070]. The views expressed are those of the authors and not necessarily those of the NIHR or the Department of Health and Social Care. This work is also supported by The Medical Research Council (MRC), The Imperial Health Charity and the NIHR Imperial Biomedical Research Centre.

Data sharing

Data are available on reasonable request

Acknowledgements

The research team would like to acknowledge all the staff members who so willingly gave their time to be interviewed.

For peer review only

Ethical approval

Relevant ethical approval was obtained from the London Harrow Ethics Committee. (17/LO/0434).

REFERENCES

[1] Bisdorff A, Von Brevern M, Lempert T, Newman-Toker DE. Classification of vestibular symptoms: Towards an international classification of vestibular disorders. J Vestib Res. 2009;19:1–13.

[2] Hartvigsen J, Boyle E, Cassidy JD, Carroll LJ. Mild traumatic brain injury after motor vehicle collisions: What are the symptoms and who treats them? A population-based 1-year inception cohort study. Arch Phys Med Rehabil. 2014;95(3 SUPPL):S286–94.

[3] Maskell F, Chiarelli P, Isles R. Dizziness after traumatic brain injury: Overview and measurement in the clinical setting. Brain Inj. 2006 Jan 1;20(3):293–305.

[4] Marcus HJ, Paine H, Sargeant M, Wolstenholme S, Collins K, Marroney N, et al. Vestibular dysfunction in acute traumatic brain injury. J Neurol. 2019;266(10):2430–3.

[5] Arshad Q, Roberts RE, Ahmad H, Lobo R, Patel M, Ham T, et al. Patients with chronic dizziness following traumatic head injury typically have multiple diagnoses involving combined peripheral and central vestibular dysfunction. Clin Neurol Neurosurg. 2017;155:17–9.

[6] Yardley L, Beech S, Weinman J. Influence of beliefs about the consequences of dizziness on handicap in people with dizziness, and the effect of therapy on beliefs. J

For peer review only

Psychosom Res. 2001;50(1):1–6.

[7] Kleffelgaard I, Langhammer B, Hellstrom T, Sandhaug M, Tamber AL, Soberg HL. Dizziness-related disability following mild–moderate traumatic brain injury. Brain Inj. 2017;31(11):1436–44.

[8] Killington MJ, Speck K, Kahlbaum J, Fabian J, Edwards D, Stobie J. Quality-of-life for individuals with a vestibular impairment following an acquired brain injury (ABI); the clients' perspective. Brain Inj. 2015 Mar 21;29(4):490–500.

[9] Chamelian L, Feinstein A. Outcome after mild to moderate traumatic brain injury: The role of dizziness. Arch Phys Med Rehabil. 2018 Jan 26;85(10):1662–6.

[10] McKechnie D, Pryor J, Fisher MJ. Falls and fallers in traumatic brain injury (TBI) rehabilitation settings: An integrative review. Disabil Rehabil. 2015;37(24):2291–9.

[11] McKechnie D, Pryor J, Fisher MJ. An examination of patient characteristics that contribute to falls in the inpatient traumatic brain injury rehabilitation setting. Disabil Rehabil. 2017;39(18):1864–71.

[12] Agrawal Y, Carey JP, Della Santina CC, Schubert MC, Minor LB. Disorders of Balance and Vestibular Function in US Adults. Arch Intern Med. 2009;169(10):938.

[13] Murphy MP, Carmine H, Kolakowsky-Hayner S. Modifiable and Nonmodifiable Risk Factors for Falls After Traumatic Brain Injury: An Exploratory Investigation With Implications for Medication Use. Rehabil Nurs. 2014 May 1;39(3):113–22.

[14] Hartholt K, van Beeck E, MD P, Polinder S, van der Velde N, MD P, et al. Societal Consequences of Falls in the Older Population: Injuries, Healthcare Costs, and Long-Term Reduced Quality of Life. J Trauma. 2011;71(3):748–53.

[15] Gerber L, Quanhong N, Hartl R, Ghajar J. Impact of falls on early mortality from severe traumatic brain injury. J Trauma Manag Outcomes. 2009;3:9.

For peer review only

[16] Carlson K, Meis L, Jensen A, Simon A, Graveley A, Taylor B, et al. Caregiver reports of subsequent injuries among veterans with traumatic brain injury after discharge from inpatient polytrauma rehabilitation programs. J Head Trauma Rehabil. 2012;27(1):14–25.

[17] Indovina I, Riccelli R, Staab JP, Lacquaniti F, Passamonti L. Personality traits modulate subcortical and cortical vestibular and anxiety responses to sound-evoked otolithic receptor stimulation. J Psychosom Res. 2018 Jan 27;77(5):391–400.

[18] Indovina I, Riccelli R, Chiarella G, Petrolo C, Augimeri A, Giofrè L, et al. Role of the Insula and Vestibular System in Patients with Chronic Subjective Dizziness: An fMRI Study Using Sound-Evoked Vestibular Stimulation. Front Behav Neurosci. 2015 Dec 9;9:334.

[19] Riccelli R, Indovina I, Staab JP, Nigro S, Augimeri A, Lacquaniti F, et al. Neuroticism modulates brain visuo-vestibular and anxiety systems during a virtual rollercoaster task. Hum Brain Mapp. 2017;38(2):715–26.

[20] Marroney N, Beattie J, Hildebrand N, Flint T, Smith RM. Does training therapists to manage benign paroxysmal positional vertigo in patients with acute traumatic brain injury reduce vestibular neurology referrals? Brain Inj. 2022 May;36(6):822–6.

[21] Hodgkinson S, Pollit V, Sharpin C, Lecky F. Early management of head injury: Summary of updated NICE guidance. BMJ. 2014;348(January):1–7.

[22] Calzolari E, Chepisheva M, Smith RM, Hellyer P, Tahtis V, Arshad Q, et al. Vestibular Agnosia in Traumatic Brain Injury and its Link to Imbalance. Brain. 2020;In Press.

[23] NHS England. NHS Standard Contract for Major Trauma Services. 2013.

[24] Rust HM, Smith RM, Mahmud M, Golding JF, Seemungal BM. Force dependency of benign paroxysmal positional vertigo in acute traumatic brain injury: a prospective study. J Neurol Neurosurg & Psychiatry. 2022 Apr 10;jnnp-2022-328997.

For peer review only

[25] Finch J. Robert Walker (ed.), Applied Qualitative Research, Gower, Aldershot, 1985. 203 pp. £17.50, paper £7.95. J Soc Policy. 2009/01/20. 1986;15(3):402–3.

[26] Creswell J. Qualitative Inquiry and Research Design: Choosing among five approaches. London: SAGE Publications; 2012. 40–66 p.

[27] Gale NK, Heath G, Cameron E, Rashid S, Redwood S. Using the framework method for the analysis of qualitative data in multi-disciplinary health research. BMC Med Res Methodol. 2013;13(1):117.

[28] Sheard L, Prout H, Dowding D, Noble S, Watt I, Maraveyas A, et al. Barriers to the diagnosis and treatment of venous thromboembolism in advanced cancer patients: A qualitative study. Palliat Med. 2012 Oct 23;27(4):339–48.

[29] Marlow LA V, McGregor LM, Nazroo JY, Wardle J. Facilitators and barriers to help-seeking for breast and cervical cancer symptoms: a qualitative study with an ethnically diverse sample in London. Psychooncology. 2014 Jul 19;23(7):749–57.

[30] Marlow LA V, Waller J, Wardle J. Barriers to cervical cancer screening among ethnic minority women: a qualitative study. J Fam Plan Reprod Heal Care. 2015 Oct 12;41(4):248–54.

[31] Shaw D, Siriwardena AN. Identifying barriers and facilitators to ambulance service assessment and treatment of acute asthma: a focus group study. BMC Emerg Med. 2014 Aug 3;14:18.

[32] Kelleher E, Harrington JM, Shiely F, Perry IJ, McHugh SM. Barriers and facilitators to the implementation of a community-based, multidisciplinary, family-focused childhood weight management programme in Ireland: A qualitative study. BMJ Open. 2017;7(8):1–10.

[33] Homer CV, Tod AM, Thompson AR, Allmark P, Goyder E. Expectations and patients' experiences of obesity prior to bariatric surgery: A qualitative study. BMJ Open.

For peer review only

2016;6(2):1–10.

[34] Gale N, Sultan H. Telehealth as 'peace of mind': embodiment, emotions and the home as the primary health space for people with chronic obstructive pulmonary disorder. Health Place. 2013;21:140–7.

[35] Rashidian A, Eccles MP, Russell I. Falling on stony ground? A qualitative study of implementation of clinical guidelines' prescribing recommendations in primary care. Health Policy (New York). 2008;85(2):148–61.

[36] Regan T, Levesque J., Lambert S., Kelly B. A qualitative investigation of healthcare professionals', patients and partners views on psychosocial issues and related interventions for couples coping with cancer. PLoS One. 2015;10:1–22.

[37] Heath G, Cameron E, Cummins C, Greenfield S, Pattison H, Kelly D, et al. Paediatric 'care closer to home': Stake-holder views and barriers to implementation. Health Place. 2012;18(5):1068–73.

[38] Ayatollahi H, Bath PA, Goodacre S. Factors influencing the use of IT in the emergency department: A qualitative study. Health Informatics J. 2010 Sep 1;16(3):189–200.

[39] French SD, Green SE, O'Connor DA, McKenzie JE, Francis JJ, Michie S, et al. Developing theory-informed behaviour change interventions to implement evidence into practice: a systematic approach using the Theoretical Domains Framework. Implement Sci. 2012;7(1):38.

[40] Michie S, Johnston M, Abraham C, Lawton R, Parker D, Walker A. Making psychological theory useful for implementing evidence based practice: a consensus approach. Qual Saf Heal Care. 2005 Feb 1;14(1):26 LP – 33.

[41] Ritchie J, Lewis J, McNaughton Nicholls C, Ormiston R. Qualitative Research Practice: A Guide for Social Science Students and Researchers. London: SAGE

For peer review only

Publications; 2013. 261–340 p.

[42] Tong A, Sainsbury P, Craig J. Consolidated criteria for reporting qualitative research (COREQ): a 32-item checklist for interviews and focus groups. Int J Qual Heal Care. 2007 Dec 1;19(6):349–57.

[43] Mañas MA, Díaz-Fúnez P, Pecino V, López-Liria R, Padilla D, Aguilar-Parra JM. Consequences of team job demands: Role ambiguity climate, affective engagement, and extra-role performance. Front Psychol. 2018;8(January):1–8.

[44] Hassan Z, Smith M, Littlewood S, Bouamra O, Hughes D, Biggin C, et al. Head injuries: a study evaluating the impact of the NICE head injury guidelines. Emerg Med J. 2005 Dec 1;22(12):845 LP – 849.

[45] Male A, Ramdharry G, Grant R, Davies RA, Beith ID. A survey of current management of Benign Paroxysmal Positional Vertigo (BPPV) by physiotherapists' interested in vestibular rehabilitation in the UK. Physiotherapy. 2018 Sep 1;105.

[46] Meldrum D, Burrows L, Cakrt O, Kerkeni H, Lopez C, Tjernstrom F, et al. Vestibular rehabilitation in Europe: a survey of clinical and research practice. J Neurol. 2020;

[47] Burrows L, Bryce K, Cole H, Haswell L, Metz D, Stevens K, et al. The ACPIVR Framework for Physiotherapists working within Vestibular and Balance System Health Care. 2021.

[48] Passi V, Johnson N. The impact of positive doctor role modeling. Med Teach. 2016 Nov 1;38(11):1139–45.

[49] Park J, Woodrow SI, Reznick RK, Beales J, MacRae HM. Observation, Reflection, and Reinforcement: Surgery Faculty Members' and Residents' Perceptions of How They Learned Professionalism. Acad Med. 2010;85(1).

[50] Polensek SH, Tusa RJ, Sterk CE. The challenges of managing vestibular disorders: a qualitative study of clinicians' experiences associated with low referral rates for

For peer review only

vestibular rehabilitation. Int J Clin Pract. 2009 Nov 1;63(11):1604–12.

[51] Newman-Toker DE, Cannon LM, Stofferahn ME, Rothman RE, Hsieh Y-H, Zee DS. Imprecision in patient reports of dizziness symptom quality: a cross-sectional study conducted in an acute care setting. Mayo Clin Proc. 2007 Nov;82(11):1329–40.

[52] Olsson Möller U, Hansson EE, Ekdahl C, Midlöv P, Jakobsson U, Kristensson J. Fighting for control in an unpredictable life – a qualitative study of older persons' experiences of living with chronic dizziness. BMC Geriatr. 2014;14(1):97.

[53] Sezier AEI, Saywell N, Terry G, Taylor D, Kayes N. Working-age adults' perspectives on living with persistent postural-perceptual dizziness: a qualitative exploratory study. BMJ Open. 2019 Apr 1;9(4):e024326.

[54] Sharp DJ, Jenkins PO. Concussion is confusing us all. Pract Neurol. 2015 Jun 1;15(3):172 LP – 186.

[55] Herdman D, Evetovits A, Everton HD, Murdin L. Is "persistent postural perceptual dizziness" a helpful diagnostic label? A qualitative exploratory study. J Vestib Res. 2021;31(1):11–21.

[56] Petty RE, Briñol P, Tormala ZL. Thought confidence as a determinant of persuasion: the self-validation hypothesis. J Pers Soc Psychol. 2002 May;82(5):722—741.

[57] Harris L, Hateley S, Tsang KT, Wilson M, Seemungal BM. Impact of anti-epileptic drug choice on discharge in acute traumatic brain injury patients. J Neurol. 2020 Jun;267(6):1774–9.

[58] von Brevern M, Zeise D, Neuhauser H, Clarke AH, Lempert T. Acute migrainous vertigo: clinical and oculographic findings. Brain. 2005 Feb;128(Pt 2):365–74.

[59] J AD, G C. Healthcare professionals in research (HPiR) Facebook community: a survey of U.K. doctoral and postdoctoral healthcare professionals outside of medicine. BMC Med Educ. 2021;21(1):236.

For peer review only

[60] Cohen HS, Gottshall KR, Graziano M, Malmstrom E-M, Sharpe MH. International survey of vestibular rehabilitation therapists by the Barany Society Ad Hoc Committee on Vestibular Rehabilitation Therapy. J Vestib Res. 2009;19(1–2):15–20.

[61] Booth J, Hewison A. Role overlap between occupational therapy and physiotherapy during in-patient stroke rehabilitation : an exploratory study Role overlap between occupational therapy and physiotherapy during in-patient stroke rehabilitation : an exploratory study. 2009;1820.

[62] Burrows L, Lesser TH, Kasbekar A V, Roland N, Billing M. Independent prescriber physiotherapist led balance clinic: the Southport and Ormskirk pathway. J Laryngol Otol. 2017/02/16. 2017;131(5):417–24.

[63] Davies P, Walker AE, Grimshaw JM. A systematic review of the use of theory in the design of guideline dissemination and implementation strategies and interpretation of the results in rigorous evaluations. Implement Sci. 2010;5:14–20.

[64] Van Bokhoven MA, Kok G, van der Weiden T. Designing a quality improvement intervention: A systematic approach. BMJ Qual Saf. 2003;12:215–20.

[65] Michie S, van Stralen MM, West R. The behaviour change wheel: A new method for characterising and designing behaviour change interventions. Implement Sci. 2011;6(1):42.

For peer review only

Figure 1. Five main themes and their relation to the overarching concept of role.

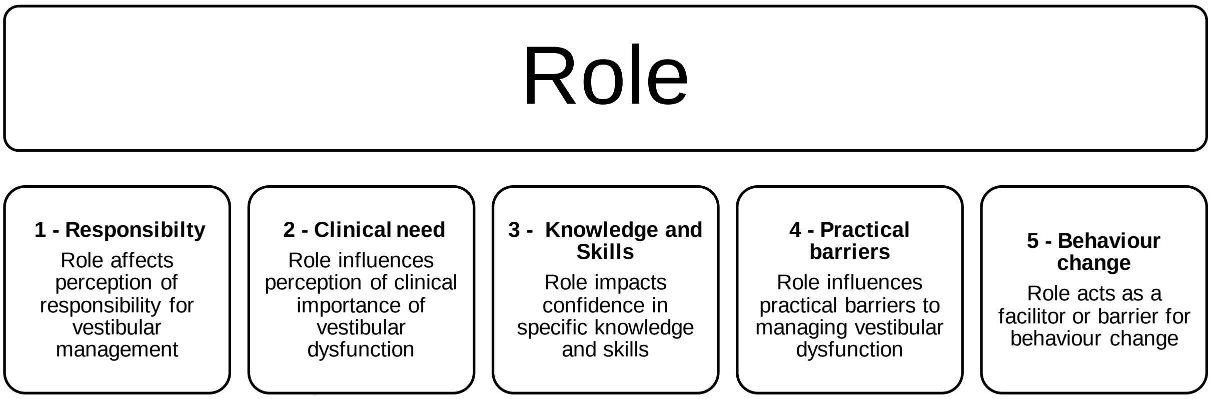

For peer review only

Figure 2. Diagram noting the themes contributing to the ‘invisibility’ of vestibular dysfunction

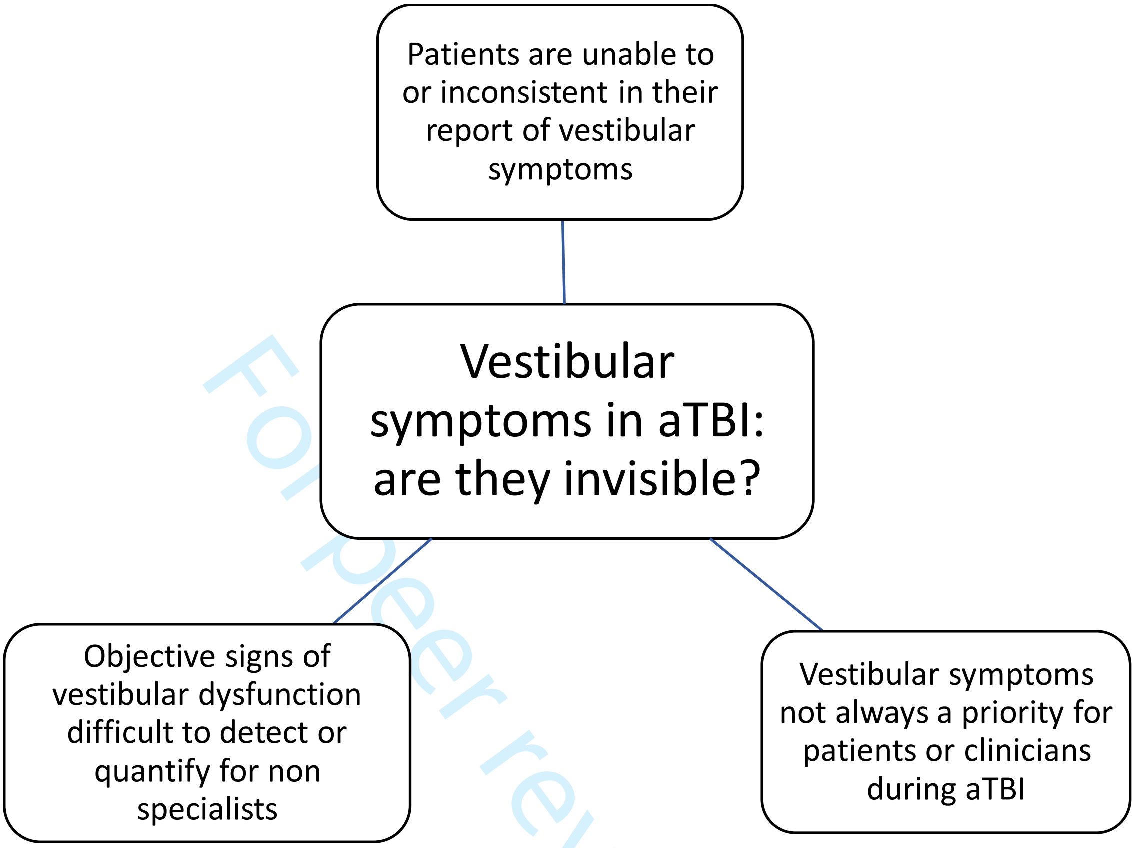

For peer review only

Supplementary Data

Supplementary File 1 - Topic guide

NHS Healthcare Professional Interview questions

Title: A qualitative study investigating the barriers and facilitators to screening of dizziness and imbalance in acute head injury patients.

Chief Investigator: Dr Barry Seemungal, Imperial College, Division of Brain Sciences, Charing Cross Hospital Campus, W6 8RF. Tel: 02033117042

Co-investigator: Rebecca Smith, Imperial College, Division of Brain Sciences, Charing Cross Hospital Campus, W6 8RF. Tel: 02033117042

<u>Introduction</u>

Thank you for agreeing to participate in this interview. The aim of the study is to explore healthcare professionals' knowledge and experience of assessing and treating dizziness in head injury patients. Your answers will be anonymous and will remain confidential. The interview will be audio recorded and I may take some notes whilst you are talking. Please keep in mind that you do not have to answer any questions you do not feel comfortable with, and we can stop the interview at any time.

<u>Background questions:</u>

1. Approximately how many patients with head injury with dizziness or imbalance do you see in a month? (i.e is dizziness a common symptom or sign after head injury?)

2. Can you talk me through the pathway of how head injury patients with dizziness or imbalance are managed?

3. Who is responsible for seeing these patients? (What do each of these team members do?)

4. What happens after they are assessed? (prompt: what is the treatment pathway? or onward referral procedure?)

We will now explore the pathway in detail using some more specific questions.

<u>Memory, attention and decision process</u>

5. Is assessing dizziness something you do routinely in head injury patients? (If not, why not?)

6. What factors might guide your decision to assess for dizziness in a head injured patient? (prompt: What clinical signs or symptoms are utilised?; formal assessments, on handover)

For peer review only

Knowledge

7. Which dizziness diagnoses are you familiar with post head injury? (Prompt: peripheral nerve injury; BPPV; migraine)

8. What sort of tests are you aware of that are useful in diagnosing dizziness? How are these test results used or interpreted? (prompt: are these standardized or validated tests?)

9. Are you aware of any treatment options for dizziness? (prompt: this might include onward referrals to other healthcare professionals)

10. Are there barriers to referring patients to appropriate services? (prompt: is there a wait for a specialist assessment?)

Skills

11. How confident are you in assessing and interpreting results of diagnostic tests for BPPV or other forms of dizziness?

12. What skills are required to treat dizziness conditions in head injury patients? (Prompt: BPPV)

Beliefs about capabilities

13. How would you rate your depth of understanding of dizziness? (prompt: How confident do you feel in managing dizziness after head injury?)

Social professional role and identity

14. Do you think conducting dizziness assessments is part of your role? If not, whose role does this fall into? (prompt: is assessing dizziness something you are trained to do?)

15. Do you think interpreting these results and providing treatment is an appropriate part of your role? Why / why not?

Beliefs about consequences

16. Are there any benefits of assessing and treating dizziness in these patients? (prompt: to yourself; patients, other clinicians; organisation).

17. Are there any consequences of 'not doing?' i.e. not assessing or treating? (prompt: is it a problem which resolves on its own; are there any consequences for the patient, clinicians, NHS, financial; long/short term) If no – why?

Motivation and goals

18. How important is it to assess and treat dizziness in head injury patients? (prompt: how high is the priority compared to other behaviours required to treat the patient?)

For peer review only

Environmental context and resources

19. Does the trauma ward situation or environment affect your management of dizzy head injury patients? (Prompt: i.e. are there time factors or competing tasks)

20. Are there clear communication channels if you are unsure of how to complete a test or interpret a result?

21. Are there any other person or environmental barriers or facilitators that might influence your screening behaviour? (prompt: guidelines/resources)

Social influences

22. Do other colleagues perform these tests? Does this influence your decision to complete / not complete these tests? (Prompt: AHPs; other medics)

23. How might the views of your colleagues affect you managing a dizzy head injured patient?

Emotion

24. Are there any situations in which you would be worried about managing dizziness in this population? (i.e medically/professionally/emotionally)

25. Are there any challenges in managing a head injury patient with dizziness?

Behavioral regulation

26. If you wanted to change your dizziness assessment behavior, how would you go about doing this? (prompt: what would facilitate you to do this?)

27. Is there any training that you or your team may require? (prompt: any procedures/guidelines/ways of working)

Lastly, is there any further you would like to add which we have not covered?

Thank you for participating in this interview.

For peer review only

Supplementary File 2 - Framework for analysis

1 Current practice

1.1 Identification and reporting of dizziness
1.2 Assessment and the assessment pathway
1.3 Treatment and referral pathway
1.4 Longer term effects of treating/not treating
1.5 Perceptions regarding role and responsibility relating to dizziness assessment and treatment
1.6 Views about specialist services

2 Dizziness and the clinical need

2.1 Factors relating to dizziness frequency, severity and longevity
2.2 Perceptions around the importance and clinical significance of dizziness
2.3 Impact of dizziness on patients
2.4 Impact of dizziness on service
2.5 Factors relating to early identification and intervention
2.6 Views about specific populations i.e. elderly or young

3 Current knowledge

3.1 Knowledge about BPPV as a condition
3.2 Knowledge and clinical reasoning relating to other causes of dizziness
3.3 Knowledge about diagnosis and treatment of BPPV
3.4 Healthcare professional's ability to diagnose and treat dizziness conditions
3.5 Efficacy of BPPV treatment

4 Barriers to diagnosis and treatment competence

For peer review only

4.1 Views about the area and motivation/interest in the area
4.2 Factors related to training, skills or knowledge
4.3 Feasibility of diagnosis and treatment
4.4 Confidence
4.5 Role concerns
4.6 Prior surgical / medical clearance
4.7 Environmental factors
4.8 Lack of access to mentors or specialists
4.9 Tangible evidence of dizziness
4.10 Views about resources – staffing, workload, time

5 Changing behaviour and practice

5.1 Factors relating to role change
5.2 Facilitators to behaviour change
5.3 Benefits of practice change
5.4 Limitations or barriers to practice change
5.5 Training requirements
5.6 Strategies for changing or improving practice
5.7 Wider translatable aspects of behaviour change

For peer review only

Supplementary data 3 – Central Chart

	Profession	Gender	Location	Current role	View on clinical need	Ax_Rx knowledge & ability	Main barrier	Readiness for change
1	Medic, FY1	Male	Trauma, Imperial	Adhoc, unspecific assessor, referrer	Low-medium	Partly theoretical, non functional	Resources & Clinical need	Reservations, not feasible to take on role
2	Medic, Registrar	Male	Surgeon, Trauma, Imperial	Inactive, reliance on therapists	Low-medium	Theoretical, partly functional	Resouces & Clinical need	Reservations, not feasible to take on role
3	Medic, FY1	Male	Trauma, Imperial	Inactive	Low-medium	Partly theoretical, non functional	1. Current role & remit 2. Resources & Clinical need	Reservations, not feasible to take on role (or just not engaged?)
4	Medic, Registrar	Female	Trauma, Imperial	Inactive; reliance on therapists	Low-medium	Non theoretical, non functional	Resources & clinical need	Reservations, not feasible to take on role
5	Medic, SHO	Male	Trauma, Imperial	Adhoc unspecific assessor, referrer	Low-medium	Non theoretical, non functional	Current role & remit	Reservations, not feasible to take on role
6	Medic, SHO	Female	Trauma, Imperial	Referrer	Low-medium	Non theoretical, non functional	Resources & clinical need	Minor reservations, not feasible to take on role
7	Medic, Registrar	Male	Trauma, Kings	Adhoc, unspecific assessor, referrer	Low-medium	Partly theoretical, non functional	Current role & remit	Reservations, not feasible to take on role
8	Medic, FY1	Female	Trauma, Kings	Adhoc, unspecific assessor, referrer	Low-medium	Partly theoretical, non functional	Resources & Clinical need	Reservations, not feasible to take on role
9	Medic, Consultant	Male	Surgeon, Imperial	Subjective/Objective identifier, unspecific assessor, referrer	Low-medium	Partly theoretical, non functional	Knowledge and skills	Reservations, not feasible to take on role
10	OT, B7	Female	Trauma, Imperial	Subjective/Objective identifier, unspecific	High	Partly theoretical, non functional	Knowledge and skills	No reservations, keen to take on role

For peer review only

				assessor and referrer				
11	OT, B6	Male	CDU, Imperial	Routine identifier, unspecific assessor, referrer	Medium-high	Partly theoretical, non functional	Current role & remit	Reservations, not feasible to take on role
12	OT, B7	Female	CDU, Imperial	Routine identifier, unspecific assessor, referrer	Medium-high	Partly theoretical, non functional	Current role & remit	Reservations, not feasible to take on role
13	OT, B8	Female	Neuro, Imperial	Subjective identifier, unspecific assessor, referrer	Medium-high	Non theoretical, non functional	Current role & remit	Some reservations, uncertain about taking on the role
14	OT, B7	Female	CDU, Imperial	Subjective identifier, unspecific assessor, referrer	High	Theoretical, non functional	Current role & remit	Some reservations, uncertain about taking on role
15	OT, B7	Female	Trauma, Kings	Routine identifier, screen, referrer	Medium-high	Theoretical, non functional	Current role & remit Confidence	Minor reservations, keen to take on role
16	OT, B7	Female	CC, Kings	Subjective identifier, unspecific assessor, referrer	Low-medium	Theoretical, non functional	Current role & remit	Minor reservations, keen to take on role
17	OT, B7	Male	CC Kings	Subjective / objective identifier, unspecific assessor, referrer	Low-medium	Partly theoretical, non functional	Current role & remit	Minor reservations, keen to take on role
18	OT, B5	Female	Trauma, Imperial	Routine identifier, unspecific assessor, referrer	Medium-high	Partly theoretical, non functional	Current role & remit Knowledge and skills	Some reservations, uncertain about taking on role

For peer review only

19	OT, B7	Female	Trauma, Imperial	Routine identifier, unspecific asessor	High	Theoretical, non functional	Current role and remit Knowledge and skills	Minor reservations, keen to take on role.
20	OT, B6	Female	Neuro, Imperial	Routine identifier, unspecific assessor, referrer	Medium-high	Partly theoretical, non functional	Current role & remit	Minor reservations, keen to take on role
21	PT, B5	Female	Neuro, Imperial	Subjective identifier, unspecific assessor	Medium-high	Partly theoretical, non functional	1. Role & Remit<br>2. Knowledge and Skills	Some reservations, uncertain about taking on role
22	PT, B7	Female	Trauma, Imperial	Routine identifier, unspecific assessor, referrer	High	Theoretical, non functional	Low confidence	Minor reservations, keen to take on role
23	PT, B6	Female	Neuro, Imperial	Subjective identifier, unspecific assessor, referrer	Medium	Partly theoretical, non functional	Knowledge and skills	Minor reservations, keen to take on role
24	PT, B7	Female	Neuro, Kings	Routine identifier, specific assessor, treat	Medium	Theoretical and partly functional	Confidence	Minor reservations, keen to take on role
25	PT, B7	Female	T & O, King's	Subjective/objective identifier, unspecific assessor, referrer	Low-Medium	Theoretical, non functional	Current role & remit	Minor reservations, keen to take on role
26	PT, B6	Male	Trauma, Imperial	Subjective/objective identifier, referrer	High	Theoretical, partly functional	Confidence	Minor reservations, keen to take on role
27	PT, B6	Female	Trauma, Imperial	Subjective/objective identifier, unspecific assessor, referrer	Medium-high	Partly theoretical, non functional		Minor reservations, keen to take on role
28	PT, B8	Female	Trauma, Imperial	Routine identifier, semi specific assessor, referrer	Medium-high	Partly theoretical, non functional	Knowledge and skills	Minor reservations, keen to take on role

For peer review only

For peer review only

For peer review only

COREQ (COnsolidated criteria for REporting Qualitative research) Checklist

A checklist of items that should be included in reports of qualitative research. You must report the page number in your manuscript where you consider each of the items listed in this checklist. If you have not included this information, either revise your manuscript accordingly before submitting or note N/A.

Topic	Item No.	Guide Questions/Description	Reported on Page No.
**Domain 1: Research team and reflexivity**			
Personal characteristics			
Interviewer/facilitator	1	Which author/s conducted the interview or focus group?	
Credentials	2	What were the researcher's credentials? E.g. PhD, MD	
Occupation	3	What was their occupation at the time of the study?	
Gender	4	Was the researcher male or female?	
Experience and training	5	What experience or training did the researcher have?	
Relationship with participants			
Relationship established	6	Was a relationship established prior to study commencement?	
Participant knowledge of the interviewer	7	What did the participants know about the researcher? e.g. personal goals, reasons for doing the research	
Interviewer characteristics	8	What characteristics were reported about the inter viewer/facilitator? e.g. Bias, assumptions, reasons and interests in the research topic	
**Domain 2: Study design**			
Theoretical framework			
Methodological orientation and Theory	9	What methodological orientation was stated to underpin the study? e.g. grounded theory, discourse analysis, ethnography, phenomenology, content analysis	
Participant selection			
Sampling	10	How were participants selected? e.g. purposive, convenience, consecutive, snowball	
Method of approach	11	How were participants approached? e.g. face-to-face, telephone, mail, email	
Sample size	12	How many participants were in the study?	
Non-participation	13	How many people refused to participate or dropped out? Reasons?	
Setting			
Setting of data collection	14	Where was the data collected? e.g. home, clinic, workplace	
Presence of non-participants	15	Was anyone else present besides the participants and researchers?	
Description of sample	16	What are the important characteristics of the sample? e.g. demographic data, date	
Data collection			
Interview guide	17	Were questions, prompts, guides provided by the authors? Was it pilot tested?	
Repeat interviews	18	Were repeat inter views carried out? If yes, how many?	
Audio/visual recording	19	Did the research use audio or visual recording to collect the data?	
Field notes	20	Were field notes made during and/or after the inter view or focus group?	
Duration	21	What was the duration of the inter views or focus group?	
Data saturation	22	Was data saturation discussed?	
Transcripts returned	23	Were transcripts returned to participants for comment and/or	

For peer review only

Topic	Item No.	Guide Questions/Description	Reported on Page No.
		correction?	
**Domain 3: analysis and findings**			
Data analysis			
Number of data coders	24	How many data coders coded the data?	
Description of the coding tree	25	Did authors provide a description of the coding tree?	
Derivation of themes	26	Were themes identified in advance or derived from the data?	
Software	27	What software, if applicable, was used to manage the data?	
Participant checking	28	Did participants provide feedback on the findings?	
Reporting			
Quotations presented	29	Were participant quotations presented to illustrate the themes/findings? Was each quotation identified? e.g. participant number	
Data and findings consistent	30	Was there consistency between the data presented and the findings?	
Clarity of major themes	31	Were major themes clearly presented in the findings?	
Clarity of minor themes	32	Is there a description of diverse cases or discussion of minor themes?	

Developed from: Tong A, Sainsbury P, Craig J. Consolidated criteria for reporting qualitative research (COREQ): a 32-item checklist for interviews and focus groups. *International Journal for Quality in Health Care*. 2007. Volume 19, Number 6: pp. 349 – 357

Once you have completed this checklist, please save a copy and upload it as part of your submission. DO NOT include this checklist as part of the main manuscript document. It must be uploaded as a separate file.

For peer review only

BMJ Open

Why are patients with acute traumatic brain injury not routinely assessed or treated for vestibular dysfunction in the UK? A qualitative study

Journal:	*BMJ Open*
Manuscript ID	bmjopen-2022-067967.R1
Article Type:	Original research
Date Submitted by the Author:	01-Dec-2022
Complete List of Authors:	Smith, Rebecca; Imperial College London, Brain and Vestibular Group, Centre for Vestibular Neurology<br>Burgess, Caroline; King's College London, School of Population Health & Environmental Sciences<br>Tahtis, Vassilios; King's College Hospital NHS Foundation Trust<br>Marsden, Jonathan; Plymouth University, School of Health Professions<br>Seemungal, Barry; Imperial College London, Brain and Vestibular Group, Centre for Vestibular Neurology,
**Primary Subject Heading**:	Neurology
Secondary Subject Heading:	Rehabilitation medicine, Qualitative research
Keywords:	Neurotology < OTOLARYNGOLOGY, TRAUMA MANAGEMENT, REHABILITATION MEDICINE, QUALITATIVE RESEARCH

SCHOLARONE™
Manuscripts

I, the Submitting Author has the right to grant and does grant on behalf of all authors of the Work (as defined in the below author licence), an exclusive licence and/or a non-exclusive licence for contributions from authors who are: i) UK Crown employees; ii) where BMJ has agreed a CC-BY licence shall apply, and/or iii) in accordance with the terms applicable for US Federal Government officers or employees acting as part of their official duties; on a worldwide, perpetual, irrevocable, royalty-free basis to BMJ Publishing Group Ltd ("BMJ") its licensees and where the relevant Journal is co-owned by BMJ to the co-owners of the Journal, to publish the Work in this journal and any other BMJ products and to exploit all rights, as set out in our licence.

The Submitting Author accepts and understands that any supply made under these terms is made by BMJ to the Submitting Author unless you are acting as an employee on behalf of your employer or a postgraduate student of an affiliated institution which is paying any applicable article publishing charge ("APC") for Open Access articles. Where the Submitting Author wishes to make the Work available on an Open Access basis (and intends to pay the relevant APC), the terms of reuse of such Open Access shall be governed by a Creative Commons licence – details of these licences and which Creative Commons licence will apply to this Work are set out in our licence referred to above.

Other than as permitted in any relevant BMJ Author's Self Archiving Policies, I confirm this Work has not been accepted for publication elsewhere, is not being considered for publication elsewhere and does not duplicate material already published. I confirm all authors consent to publication of this Work and authorise the granting of this licence.

For peer review only

Why are patients with acute traumatic brain injury not routinely assessed or treated for vestibular dysfunction in the UK? A qualitative study

Rebecca M Smith[1†], Caroline Burgess[2], Vassilios Tahtis[1,3] , Jonathan Marsden[3], Barry M Seemungal[1†]

1. Brain & Vestibular Group, Centre for Vestibular Neurology, Imperial College London
2. School of Population Health & Environmental Sciences, King's College London
3. King's College Hospital NHS Foundation Trust
4. School of Health Professions, University of Plymouth

†Corresponding authors

10L14 Laboratory Block

Charing Cross Campus

London

W6 8RP

Email: Rebecca.smith@imperial.ac.uk; b.seemungal@imperial.ac.uk

For peer review only

ABSTRACT

Objectives

Vestibular dysfunction is common in patients with acute traumatic brain injury (aTBI). Persisting vestibular symptoms (i.e. dizziness and imbalance) are linked to poor physical, psychological, and socioeconomic outcomes. However, routine management of vestibular dysfunction in aTBI is not always standard practice. We aimed to identify and explore any healthcare professional barriers or facilitators to managing vestibular dysfunction in aTBI.

Design

A qualitative approach was used. Data were collected using face to face, semi-structured interviews and analysed using the Framework approach.

Setting

Two Major Trauma Centres in London, UK.

Participants

28 healthcare professionals participated: 11 occupational therapists, 8 physiotherapists and 9 surgical/trauma doctors.

Results

Vestibular assessment and treatment was not routinely undertaken by trauma ward staff. Uncertainty regarding responsibility for vestibular management on the trauma ward was

For peer review only

perceived to lead to gaps in patient care. Interestingly, the term dizziness was sometimes perceived as an ‘invisible’ and vague phenomenon, leading to difficulties identifying or ‘proving’ dizziness and a tendency for making non-specific diagnoses. Barriers to routine assessment and treatment included limited knowledge and skills, a lack of local or national guidelines, insufficient training, and concerns regarding the practical aspects of managing vestibular dysfunction. Of current trauma ward staff, therapists were identified as appropriate healthcare professionals to adopt new behaviours regarding management of a common form of vestibular dysfunction (benign paroxysmal positional vertigo). Strategies to support this behaviour change include heightened clarity around role, implementation of local or national guidelines, improved access to training, and multidisciplinary support from experts in vestibular dysfunction.

Conclusions

This study has highlighted that role and knowledge barriers exist to multidisciplinary management of vestibular dysfunction in aTBI. Trauma ward therapists were identified as the most appropriate healthcare professionals to adopt new behaviours. Several strategies are proposed to facilitate such behaviour change.

For peer review only

Article Summary

Strengths and limitations of the study

- This multi-centre qualitative study is the first to explore barriers and facilitators to managing vestibular dysfunction in aTBI in a range of healthcare professionals.
- Data were analysed using a systematic, transparent approach, framework analysis, to heighten rigour and trustworthiness within the results.
- Whilst the study size was modest, healthcare professionals were purposefully sampled to encompass a range of professional backgrounds and experience.
- Although patients were recruited from two sites, this represents half of the major trauma centres in London, UK, supporting the transferability of findings to other urban major trauma centres in the UK.
- This study investigates healthcare professionals' barriers and facilitators to managing vestibular dysfunction in aTBI, future work could usefully explore patients' and carers' experiences.

Word count: 5297

For peer review only

INTRODUCTION

Vestibular dysfunction in TBI – linked to injury of peripheral (i.e. inner ear and nerve) or central (i.e. brain) vestibular structures can result in dizziness or imbalance [1] and is very common, affecting up to 80% of ambulant moderate-to-severe acute TBI patients (aTBI) [2,3]. Vestibular dysfunction in aTBI may be caused by a range of diagnoses including benign paroxysmal positional vertigo (BPPV), centrally mediated gait ataxia (typically a 'vestibular ataxia'), migraine phenotype headache, and acute peripheral unilateral vestibular loss [4]. Patients typically present with multiple vestibular diagnoses [4,5], increasing the complexity of assessment and treatment and elevating the risk of missed diagnoses.

Early management of vestibular dysfunction following TBI appears to be important. Delays to or inaccurate diagnosis and treatment may adversely impact patients' physical and psychosocial outcomes and quality of life [6–8], whilst persisting vestibular symptoms have been found to delay return to work [9]. Vestibular dysfunction, by its link to falls [10–12] (which affect half of TBI survivors [13]), results in significant physical, psychological and healthcare costs [14–16]. Evidence also points to the direct impact of vestibular dysfunction on mental wellbeing, with studies reporting links between the vestibular system and brain areas involved in emotional and cognitive processing [17–19]. Despite the need for early intervention, routine vestibular assessment and treatment during aTBI does not appear to be commonplace. Previous studies noted long delays to diagnosis and treatment [8], and large discrepancies in assessment and treatment practices between trauma centres [20]. Potential explanations for a lack of routine vestibular management in aTBI include: (i) limited ward based vestibular expertise (ii) absence of recommendations for vestibular assessment or treatment in national early management of head injury guidelines [21] and (iii) a newly described but common clinical phenomenon called vestibular agnosia (blunting of vestibular perception in patients with moderate-severe aTBI) [22].

For peer review only

The lack of vestibular expertise on major trauma wards is perhaps not surprising since the complexity of vestibular dysfunction has only recently been elucidated [4,5,22], and vestibular neurologists / specialists do not appear in the current list of recommended trauma ward based healthcare professionals [23]. Whether the latter is a consequence of or contributing factor to national guidelines not stipulating the need of routine vestibular assessment or management is unclear, however the two factors may well be interdependent. Thus, major trauma wards are often staffed by surgical specialities who are expert at acute life-saving interventions but less so at managing the neurological complications of TBI (dizziness, imbalance, and cognitive sequelae). Perhaps challenging for healthcare professionals is the finding that vestibular dysfunction in aTBI, with its attendant increased risk of falls, can be 'silent' because of disrupted vestibular perception causing a vestibular agnosia (linked to a seven-fold reduction in recognition of common diagnoses such as BPPV by ward staff) [22]. Traditional teaching for healthcare professionals is to perform a focussed examination based upon the history. However, in aTBI, the more significant the brain injury the higher the underlying vestibular burden, but the less likely the patient is to report symptoms. For example, BPPV is twice as common in aTBI with skull fracture than in patients without skull fracture; i.e. 33% vs. 66% [24]. The combination of a very high vestibular burden in aTBI, its silent nature, and the standard approach to perform symptom-specific examination results in many patients being discharged home without any vestibular diagnoses, let alone specific treatment.

In summary, although early vestibular assessment and treatment in all aTBI patients is warranted, it is not routinely implemented. Prior to any change in policy or practice, it is imperative to understand the views of those routinely delivering the service or those who are likely to be affected by it [25]. Accordingly, we aimed to explore healthcare professional barriers or facilitators associated with screening, assessment, and treatment of vestibular dysfunction in aTBI patients.

For peer review only

MATERIALS AND METHODS

Patient and public involvement

Patients were involved in aiding identification and prioritisation of the research question and study design. More specifically, during an information session, patients from a local TBI association reported sub optimal experiences of vestibular dysfunction management in aTBI, whilst healthcare professionals were perceived to poorly understand symptoms of vestibular dysfunction. Such feedback formed the basis of the present study.

Study Design

A qualitative methodology, the Framework approach, was used to gather experiential data [26,27]. Originally this methodology was developed for large scale policy research, but has been utilised more widely in health research [28] and pertinently, in studies exploring barriers to diagnostic and treatment implementation [29–32].

Sampling, Sample Size and Participants

Clinicians were invited by email to participate if (i) they routinely worked on a trauma, emergency, rehabilitation, or other ward receiving patients directly from acute trauma wards, and (ii) had a role in routine assessment and treatment of aTBI patients. Purposive sampling was used to obtain a sample of healthcare professionals with differing levels of experience. Guidance from previous studies using Framework analysis and discussions regarding data saturation were used to determine an appropriate sample size [33,34]. This sampling method is in line with similar studies [35,36].

Data collection and setting

Semi-structured, individual, face-to-face interviews were conducted by the same researcher (RS), using a topic guide (Supplementary file 1) to gather in-depth data [37–39]. Interviews

For peer review only

were audio-recorded and transcribed verbatim. Written consent was obtained from all participants.

The theoretical domains framework (TDF) was utilised to inform the topic guide. The TDF was used to (i) allow a greater understanding of factors influencing clinical behaviour, (ii) determine possible strategies to change behaviour, and (iii) clarify how such strategies might be best executed [40]. The TDF was developed to identify psychological and organisational theory relevant to health practitioner behaviour change; culminating in 12 domains covering factors such as knowledge, skills and social and professional roles [41]. The TDF may therefore provide a theoretical model for any subsequent behaviour change intervention, and further, may enable successful implementation of that intervention. The topic guide included questions on how symptoms of vestibular dysfunction i.e. dizziness and imbalance were managed on the trauma ward. Prompts and probes were utilised to stimulate further discussion, if required [42]. This approach has also been used in other studies employing the TDF and Framework analysis [33,43]. Participants were not asked to define dizziness or imbalance as this did not form part of the primary research question.

Data analysis

Data were analysed utilising the Framework approach; a series of interconnected stages enabling the researcher to move back and forth across the data until a coherent account emerges. During analysis the data is charted, and sorted according to key themes [44]. Two researchers reviewed and refined the framework (Supplementary file 2), which underwent several iterations before it encompassed the whole data set. Using NVivo (v.12), the data were charted, whereby indexed data were summarised for each participant. After charting ten transcripts, data were discussed amongst the research team. Some subthemes were noted to be redundant or hold significant overlap and were removed or renamed. Following refinement of the framework, the final transcripts were charted.

For peer review only

Mindmaps (MindView v.7) were created for each of the five themes, providing a visual representation of the whole data set. Notes were made on connections, patterns and areas of convergence and divergence between participants, subthemes, or themes. Finally, a central chart (Supplementary file 3) was created encompassing all respondents across both sites. This was used to explore patterns across themes and participant groups.

RESULTS

Results are reported in accordance with Consolidated criteria for reporting qualitative research (COREQ) guidelines where possible [45]. Thirty-five healthcare professionals across two Major Trauma Centres were invited to take part: of those, seven declined to take part or did not reply to the invitation email and 28 participated. Those declining to take part were evenly spread regarding their profession and level of experience. Table 1 illustrates the demographics of the 28 participants. In our sample, healthcare professionals had on average 47.4 months experience of working in trauma.

Healthcare Professional	Number interviewed	Female	Speciality (number)
Junior Therapist	7	5	Physiotherapist (4)<br>Occupational Therapist (3)
Senior Therapist	12	10	Physiotherapist (4)<br>Occupational Therapist (6)
Junior doctor	5	2	Surgery (3)<br>Trauma (2)
Senior doctor (Registrar-Consultant)	4	1	Neurosurgery (2)<br>Trauma (2)

Table 1. Demographics of study participants.

For peer review only

Five main themes relating to healthcare professionals' experiences of managing vestibular dysfunction are outlined; (i) Current practice – who is responsible for screening, assessing, and treating vestibular dysfunction? (ii) The invisibility of dizziness: how clinically important is it? (iii) How confident are healthcare professionals in their knowledge and skills to assess and treat vestibular dysfunction? (iv) What are the practical barriers to assessment and treatment? and (v) Who and what is required for behaviour change? These five main factors were noted to be connected by an overarching characteristic: Healthcare professionals' role (Figure 1). Quotes from participants are included to illustrate each of the five main themes. Quotes are followed by the pseudonym, profession, and speciality of the participant.

Figure 1 here

Figure 1. Five main themes relating to healthcare professionals' experiences of managing vestibular dysfunction and their relation to the overarching concept of role.

Current practice – who is responsible for screening, assessment, and treatment of vestibular dysfunction?

Trauma and surgical doctors across both sites felt a theoretical sense of oversight for all trauma related deficits, however there was uncertainty regarding responsibility for day-to-day management of vestibular dysfunction. Doctors appeared less likely to ask about dizziness or assess vestibular dysfunction routinely. However, one divergent case, a consultant neurosurgeon, reported screening patients more frequently although this was guided by the presence of skull fractures on a scan, rather than a routine question.

For peer review only

'No, we absolutely don't assess it routinely… it's not something that we directly asked questions about, so we often missed it'

[A13, Registrar, Surgery]

Therapists (physiotherapists and occupational therapists) also expressed uncertainty about responsibility for managing vestibular dysfunction, although in practice they tended to identify patients with vestibular dysfunction and coordinate referrals. Therapists attributed taking on these roles due to spending more time with patients and being the first healthcare professional to mobilise or complete functional tasks with patients (these activities often provoked dizziness). Large variability was noted in how therapists identified patients; some routinely asked about dizziness or imbalance, whereas others relied on patient report or manifestations of vestibular dysfunction in patients' balance, gait, or body language. Following identification, therapists referred patients to specialists. No therapists at either site reported completing specific assessments or treatments independently. There was a sense amongst occupational therapists in particular, that managing vestibular dysfunction was outside of their remit and instead embedded in a physiotherapist's role (due to their existing involvement in balance assessment).

'I think when you're an OT, probably the expectations were that you're not the one doing that. I would have no problem to do it if I felt that I was trained to do it. I think it's just never been something I've ever been encouraged to look into because, generally, the physios come along and do it…I think it might be a cultural thing within therapy'

[A6, Occupational therapist, Senior Specialist]

For peer review only

Respondents reported variability concerning when and to whom patients were referred. At both sites, specialists were visiting teams and therefore not regularly ward or sometimes even site based, leading to variation in time-to-assessment and occasionally patients being discharged prior to assessment. Interestingly, although specialist input was universally noted to be positive, respondents also viewed the presence of specialists as a barrier to improving their own vestibular knowledge and skills. Uncertainty was also evident in which patients were eligible for follow up post-discharge. Therapists appeared to devolve responsibility to patients to contact their General Practitioner if they had ongoing symptoms of vestibular dysfunction. One participant described this could lead to patients 'falling through gaps'. The uncertainty around responsibility and the variability noted in treatment pathways were thought to have a negative impact on vestibular care during and following admission.

> *'I don't know about a clear pathway, there's not really a protocol I should say, it's bit more adhoc…The service is not really equitable for everybody, and also probably people are lost to follow up and have poor outcomes in the future'*
>
> [A5, Physiotherapist, Senior]

The invisibility of dizziness: How clinically important is it?

There were marked differences between doctors and therapists regarding their perception of the clinical importance of dizziness, seemingly related to expectations of their role. Doctors perceived dizziness as a short-lived symptom and where it did persist, it could be managed in outpatient rather than inpatient settings.

> *'Not a massive priority…I think the feeling is that if someone has a head injury, they probably have a bit of, they could well have some dizziness, but it may not you know, it'd probably just be transient'*
>
> *[A27, Registrar, Trauma]*

For peer review only

Doctors’ views on the importance of dizziness appeared to be related to their clinical priorities of immediate life preservation or signs of acute deterioration. As one participant described ‘we often take our foot off the pedal a little bit and dismiss other things as unimportant’. This seemingly manifested in how frequently they asked about vestibular symptoms. Doctors noted the impact of dizziness on patients’ confidence and balance, but generally were more ambivalent about it causing direct harm. Notably there was the feeling that it could delay discharge.

> *‘These patients are not so bad that they have to be seen within 24 hours, even if you delayed it [treatment] by two or three days it’s not a big issue. It increases their hospital stay but it doesn’t cause any harm to them, so it’s not a big problem’*
>
> *[A8, Consultant, Neurosurgeon]*

Interestingly, ‘subtle’ or ‘invisible’ were words used to describe dizziness, attributed to ward round assessments being conducted whilst patients were lying still when signs of vestibular dysfunction were not always apparent (to a non-expert) and/or dizziness was not reported. Further, respondents noted the subtlety, subjective and positional nature of dizziness was not only a barrier to identification (and therefore accurate assessment and treatment), but also in ‘proving’ patients were dizzy, which was further seen to limit its clinical importance. Respondents did not mention the potential for objective measurement of vestibular dysfunction and hence were likely unaware of the capability for definitive diagnosis via laboratory testing.

> *‘It’s not seen on a scan and so often it’s the importance of it is hard to emphasise to the wider sort of medical community so whereas if it’s a blood result you can show it and I think the impact is probably underestimated. As physiotherapists we probably*

For peer review only

know the impact of it but generally, I'm not convinced the wider medical community recognises the repercussions.'

[A3, Physiotherapist, Senior]

Therapists viewed dizziness as a higher priority; increasing risk of falls, impacting cognition, attention, confidence, independence, and emotional and social wellbeing. Additionally, dizziness was noted to impede progress with recovery, resulting in an increased length of stay, heightened demand on ward therapy staff, and more support at home.

'If they're feeling dizzy, they spend longer periods in bed and they're up and walking around less, which then obviously has a lot of other secondary complications in terms of prolonged bed rest and not moving around and decreased appetite or decreased oral intake, just because they're struggling to get up. And then I think it can increase their length of stay"

[A11, Physiotherapist, Senior Specialist]

How confident are healthcare professionals in their knowledge and skills to assess and treat vestibular dysfunction?

Across all respondents, there was some theoretical knowledge but limited ability or confidence with practical vestibular assessment and treatment skills. Therapists denied knowledge or use of vestibular assessment tools, whilst there was wider awareness and use of 'balance measures' and assessment for postural hypotension. Low confidence was noted in undertaking eye movement examinations, and interpretation of findings was felt to be out of the scope of their knowledge and skills.

'I don't routinely do an actual dizziness assessment. I'd maybe look into balance and see whether they've got poor balance which might be linking everything in... I don't have a specific assessment to do'

For peer review only

[A10, Physiotherapist, Junior]

Respondents exhibited some theoretical knowledge of how to undertake clinical bedside diagnostic and treatment manoeuvres for the most common cause of peripheral vestibular dysfunction (BPPV), but little or no confidence in practical skills. Where there was practical experience, this was limited to physiotherapists who were not routinely treating patients due to (i) low confidence secondary to limited patient exposure and insufficient training and mentorship, (ii) a reliance on visiting specialists, or (iii) the practicality of undertaking treatment in aTBI patients. Across both sites therapists had little confidence in other therapy or medical staff to manage BPPV. Therapists noted specialists were effective at treating BPPV, although there was divergence regarding dosing and optimum time to treat.

> *'I don't think any of us up here feel confident to do it…I also think because the [trauma] doctors are rotational and don't necessarily have in-depth knowledge, they won't be confident to prescribe or treat. They definitely don't know how to do the manoeuvres'*

[A5, Physiotherapist, Senior]

Similarly, trauma or surgical doctors did not report use of specific vestibular assessments. There was more confidence in completing general eye movement examinations, although interpretation of findings and discerning peripheral (i.e. inner ear) versus central (i.e. brain) patterns was felt to be complex. Some theoretical knowledge of the BPPV diagnostic test was apparent, but there were lower levels of confidence in their (or their therapy or medical colleagues) ability to practically undertake bedside tests or treatment. Non-specific treatments such as medication to supress vertigo were most frequently reported as first-line treatment.

For peer review only

‘I don’t think anyone in my team including myself would confidently say we can deal with it…I don’t think the therapists would go to the extent of actually doing specific manoeuvres, the Hallpike manoeuvre’

[A8, Consultant, Neurosurgeon]

What are the practical barriers to assessment and treatment of vestibular dysfunction?

Participants perceived training and knowledge as the most fundamental barrier to managing vestibular dysfunction. Secondary barriers were both intrinsic (motivation and confidence) and extrinsic (time and the feasibility of completing diagnostic and treatment procedures). Whilst both physiotherapists and occupational therapists felt their previous vestibular training did not enable them to assess and treat independently, occupational therapists perceived their training was not comparable to that of physiotherapists. Although neither physiotherapists nor occupational therapists felt managing vestibular dysfunction was an expectation of their role, occupational therapists felt concerned that taking an interest would cross professional boundaries. Dizziness and imbalance were still perceived to be relevant to their role, however.

‘I’m not sure whether we would be um sort of stepping in some area that is not supposed to be ours however in terms of occupational therapy, it’s something that affects function so I think it’s relevant…we should be more aware of how to treat’

[A4, Occupational therapist, Junior]

For peer review only

For those receiving more training, limited or adhoc exposure to patients and hence reduced application of practical skills, coupled with the rotational nature of training seemingly affected confidence, knowledge, and skill consolidation.

> *'I haven't actually been taught how to do it [assessment and treatment of BPPV], I've just been shown it, or told the basis behind it, rather than actually being taught to carry it out…I think rotating out of it all the time and just not, never really having the chance to consolidate skills… again it's not really something that we're expected to manage'*
>
> *[A11, Physiotherapist, Senior Specialist]*

Doctors recalled some undergraduate teaching but felt this was not revisited during clinical training, perhaps associated with their focus on managing acute aspects of TBI. Further, there was a perception amongst doctors at both sites of dizziness as an ill-defined symptom, without discrete cause or diagnosis and without specific treatment. This view of dizziness as 'unfixable' seemed particularly important for neurosurgeons who were accustomed to being able to 'fix' symptoms. Interestingly, this perception was not noted amongst therapists.

> *'There is this concept of post-concussion dizziness where it's really non-specific and the idea is it's not really treatable, it's not a specific condition. And so if it's not treatable and particularly as surgeons our mindset is to only think about things you can fix…I think internationally that post-concussion dizziness isn't discrete diseases, it's just a fluffy phenomenon that occurs as a sequelae of head injury'*
>
> *[A13, Registrar, Surgery]*

For peer review only

Time constraints were particularly evident amongst doctors and therapists working in emergency areas, seemingly due to competing priorities. Contrastingly, trauma therapists highlighted two secondary barriers (i) paucity of role models (ii) feasibility of identifying vestibular dysfunction and performing assessments and treatments in aTBI patients due to spine and limb fractures, pain, consciousness, communication, cognition, and insight.

Who and what is required for behaviour change?

When asked about incorporating managing vestibular dysfunction into their role, trauma therapists displayed the most enthusiasm about behaviour change. This was corroborated by doctors, who perceived therapists to have adequate time and to see patients at an appropriate point in their recovery. Trauma therapists were specific about exclusively managing BPPV, rather than other causes of vestibular dysfunction.

> *'If I had the right training, I'd be very happy to go and treat, considering that it's [BPPV treatment] almost 100% effective and its quicker turnaround, absolutely. I don't think, why would we need to waste more resources and time really? Because what am I just screening in order to get someone else to come and fix the problem? So, it might be better if we were trained, I think it would be in our scope'*
>
> *[A25, Occupational therapist, Senior Specialist]*

Reservations to behaviour change included (i) concerns regarding remit (occupational therapists) and (ii) concerns around staff capacity and transferability of skills (physiotherapists). Facilitators involved changes to local or national guidelines and accessible information for healthcare professionals' and patients to help consolidate awareness and knowledge.

"Some sort of policy or practice because I don't think it's enough just to do some training. Because I think people go for these trainings, then just don't do it. I think has to maybe come from something bigger, like a change in policy…. or something locally like a policy on head injury management or vestibular management"

[A7, Physiotherapist, Team Lead]

Role-related facilitators included setting clear expectations, regular patient exposure, vestibular neurology team support for complex cases, training and mentorship, and endorsement from line managers. Heighted theoretical and practical training and training a range of therapists to ensure sufficient capacity and maintenance of skills was felt necessary. A theoretical and practical checklist was thought to improve confidence and ensure consistency. Potential benefits to behaviour change included more timely assessment and treatment, shortened hospital stay, improved progress with therapy and fewer patients with BPPV being missed.

"I think it would only benefit the patient because actually earlier on we'd be focusing on all aspects and picking it up better... And I think as well if you can settle someone's dizziness earlier on, they'll engage better with the therapy along the way because actually if they're always dizzy and we're struggling with that for a while and we're waiting for that assessment and then the treatment, you know you want the patient to build confidence"

[A26, Physiotherapist, Senior Specialist]

For peer review only

DISCUSSION

Summary of findings

This study provides new insights into the impact of healthcare professional's perceived role on vestibular assessment and treatment behaviours in aTBI. Our findings suggest management of vestibular dysfunction may be affected by (i) uncertainty within healthcare professionals' role and assessment and treatment pathways, (ii) self-reported lack of confidence in practical skills, (iii) lack of training, access to a multi-disciplinary team for complex cases, and mentorship and (iv) a perception of dizziness as an invisible and un-fixable entity. Optimism was expressed however, towards the possibility of behaviour and role change to improve the care pathway.

The role-related uncertainty displayed by healthcare professionals may be linked to lack of detail in existing guidelines in how, when and by whom post-traumatic vestibular dysfunction should be managed [21,23]. Previous research notes sufficient clarity surrounding role is significant in relation to completing and focusing on important tasks [46], whilst implementation of guidelines in other areas of aTBI management noted improved practice and cost savings [47]. Thus, heightened clarity around clinicians' roles and formation of a care pathway or guidelines may be useful in improving care for aTBI patients with vestibular dysfunction.

Insufficient training and a scarcity of role models were also perceived to be barriers to routine care. The degree to which vestibular management is included in undergraduate medical or therapy training is unclear, however published surveys note training is variable and appears to be undertaken at postgraduate level [48,49]. To date there are no formal qualifications for therapists involved in managing general vestibular dysfunction although draft proposals are in process for physiotherapists [50]. Such subspeciality training could

For peer review only

involve integration with a larger infrastructure providing access to expert review and audit of complex cases, training, research opportunities and mentorship. The lack of available role models noted by our participants is also noteworthy given the documented benefits of clinical behaviour, identity and career development [51,52]. Indeed, the absence of role models may have contributed to the ambiguity around responsibility for patient care and thus would be important to address for behaviour change.

Our participants described the term dizziness as an invisible or subtle phenomenon. Previous research corroborates this, noting (i) healthcare professionals perceive dizziness to be a vague symptom [53]; (ii) patients themselves exhibit inconsistencies in subjective reporting of dizziness [54] and (iii) when patients were asked about their perceptions of living with dizziness, 'invisible' was commonplace [55,56]. Importantly, these latter studies noted patients with chronic dizziness associated invisibility with a lack of self-validation and validation from healthcare professionals, thus giving weight to the rationale for early diagnosis and treatment in aTBI patients. Dizziness was described as 'subtle' by participants in our study, despite objective signs of vestibular dysfunction being elicitable (albeit by experts) in the majority of aTBI patients [4,24]. Given many patients complain of few or no vestibular symptoms (due to vestibular agnosia) [22], it is perhaps not surprising that aTBI patients' perceived dizziness is indeed, subtle (Figure 2). It follows that trauma and surgical doctors view of dizziness as a non-specific entity, without need for further diagnosis or specific treatment is understandable, although incorrect given the latest research [4,22,24]. Indeed, the general term 'post-traumatic dizziness' has historically been used in literature and practice rather than specific diagnoses, although recent work highlights the need to diagnose discrete conditions [4,5,57]. A specific diagnosis is important for accurate treatment and for self-validation [56,58], which in turn can influence attitudes and beliefs [59]. Further high-quality treatment studies may encourage the use of specific vestibular

For peer review only

diagnoses amongst healthcare professionals, thus providing patients with accurate treatment and validation of their symptoms.

Figure 2 here

Figure 2. Diagram noting the themes contributing to the 'invisibility' of vestibular dysfunction

Surgical and trauma ward doctors assumed oversight for trauma patient care, however due to their focus on life-threatening complications of aTBI and the specialist nature of managing vestibular dysfunction, they tended to feel managing dizziness and imbalance were better suited to visiting specialists. Therefore, it is perhaps not practical to expect surgical or trauma doctors to acquire the necessary competency to manage vestibular dysfunction, and thus new models of care should incorporate clinicians who either already were sufficiently trained or had capacity to be trained and mentored. From our sample trauma therapists (physiotherapists and occupational therapists) felt most able to adopt new assessment and treatment behaviours, limited to managing BPPV rather than all causes of post-traumatic vestibular dysfunction. This is unsurprising as vestibular presentations may interact with other complex post-traumatic neurological conditions such as epilepsy, where some treatments may worsen dizziness and imbalance and delay discharge [60], or vestibular migraine whose commonest acute manifestation is gait ataxia [61]. An optimal scenario may involve a team; therapists trained, supported, and mentored by a clinician, such as a vestibular neurologist, who is also capable of managing complex vestibular presentations, allowing the provision of a comprehensive aTBI neurological service. National and/or local guidelines delineating which cases would benefit from this service would improve management within the aTBI cohort. Useful strategies may additionally include formation of a therapy led group from UK major trauma centres with the aim of developing peer support and sharing research, experiences, and training. Such strategies have been found to be

For peer review only

effective in other areas [62]. There is a precedent for physiotherapists taking on vestibular roles, however few occupational therapists appear to practice in this area in the UK [20]. This trend is not limited to TBI, as a majority of physiotherapists work with vestibular patients in other albeit mainly outpatient areas [49,63]. However, the roles physiotherapists and occupational therapists undertake can and do overlap, as noted by studies of inpatient stroke rehabilitation where such overlap was found to benefit patient care [64]. Inpatient, multidisciplinary settings such as trauma centres, could therefore offer an environment where overlap of roles may provide additional staff capacity and support (a highlighted concern of respondents in our study). Acknowledging any reservations and working with all healthcare professionals involved would be key to improving future care pathways. Whether changes in behaviour would improve patient and service pathways remains unclear, however evidence in other clinical settings suggests therapist led treatment can reduce referrals, hospital visits [65], and patients' falls risk [66,67].

Successful behaviour change requires a validated and well-designed intervention [68,69], preferably designed using a framework to ensure relevant factors are considered [68]. Using the Behaviour change wheel [70], suggestions for appropriate intervention functions and associated policies are shown in Table 2.

Essential condition	Intervention	Policies	BPPV specific strategy
Capability	Education	Communication	Providing examples of therapists managing BPPV in other clinical areas
		Guidelines	National or local guidelines recommending vestibular assessment and treatment in acute TBI
	Training	Communication	Regular teaching on theoretical and practical assessment and treatment techniques for BPPV
Opportunity	Enablement	Service provision	Ensuring therapy teams have sufficient clinical capacity, role models and managerial support

For peer review only

		Regulation	Medical (Consultant level) support for complex cases<br><br>Senior therapists being seen to use skills and train junior therapists<br><br>Making relevant teams and individuals aware of a change in practice and a change in role
	Modelling	Communication	Clear expectations of what the role would involve
		Regulation/Guidelines	Embedding a pathway of care for patients with vestibular diagnoses in TBI into acute services<br><br>Data showing which patients would benefit from assessment and treatment
Motivation	Persuasion	Communication	Using data to show patient and service level benefits of assessing and treating patients early

Table 2. Table showing possible behaviour change interventions for therapists

Limitations

Although this was a multi-centre study, the two participating sites were from a similar locality, and thus the generalisability of our findings may be limited. Whilst the participants sampled had a range of experience, we recruited only therapists and trauma and surgical doctors. Specialist brain injury nurses could have also been sampled, however previous literature notes therapists and doctors are most commonly involved in managing ward patients with vestibular dysfunction [49]. Notably, the involvement of neurologists with vestibular expertise in assessing aTBI – as occurs at our Trust - is uncommon in the UK. Finally, we did not ask participants to define dizziness at the outset of interviews. This may have resulted in slight subjective differences in participants' meanings of the term dizziness and should be considered when interpreting findings.

For peer review only

CONCLUSIONS

This multi-centre qualitative study highlights the barriers and facilitators to providing timely and accurate care for aTBI patients with vestibular dysfunction. A range of role, knowledge, and practical barriers to managing vestibular dysfunction in aTBI were noted. Within our sample, trauma therapists appeared most suited to incorporate new assessment and treatment behaviours for BPPV into routine practice, supported by an appropriately trained multidisciplinary team. Theory based strategies for implementing interventions to change behaviour are proposed. Further work is required to establish whether such changes in behaviour would result in patient and/or service level improvements.

Author Contributions

All authors contributed to this work. RS designed/conceptualised the study/collected data/analysed data and drafted the manuscript. RS holds a MRes and is an NIHR Doctoral Fellow and a physiotherapist by background. CB designed/conceptualised the study/analysed data and revised the manuscript. VT designed and conceptualised the study and revised the manuscript. JM designed/conceptualised the study and revised the manuscript. BS designed/conceptualised the study/revised the manuscript and supervised the study.

Competing interests

RS, CB, VT and JB declare no competing interests. BMS is an ABN traumatic brain injury advisory committee member, a NICE guidelines review committee for head trauma and Editor in Chief for Journal of Concussion

Funding statement

For peer review only

Rebecca Smith is funded by the National Institute of Health Research [ICA-CDRF-2017-03-070]. The views expressed are those of the authors and not necessarily those of the NIHR or the Department of Health and Social Care. This work is also supported by The Medical Research Council (MRC), The Imperial Health Charity and the NIHR Imperial Biomedical Research Centre.

Data sharing

Data are available on reasonable request

Acknowledgements

The research team would like to acknowledge all the staff members who so willingly gave their time to be interviewed.

Ethical approval

Relevant ethical approval was obtained from the London Harrow Ethics Committee. (17/LO/0434).

REFERENCES

[1] Bisdorff A, Von Brevern M, Lempert T, Newman-Toker DE. Classification of vestibular symptoms: Towards an international classification of vestibular disorders. J Vestib Res. 2009;19:1–13.

For peer review only

[2] Hartvigsen J, Boyle E, Cassidy JD, Carroll LJ. Mild traumatic brain injury after motor vehicle collisions: What are the symptoms and who treats them? A population-based 1-year inception cohort study. Arch Phys Med Rehabil. 2014;95(3 SUPPL):S286–94.

[3] Maskell F, Chiarelli P, Isles R. Dizziness after traumatic brain injury: Overview and measurement in the clinical setting. Brain Inj. 2006 Jan 1;20(3):293–305.

[4] Marcus HJ, Paine H, Sargeant M, Wolstenholme S, Collins K, Marroney N, et al. Vestibular dysfunction in acute traumatic brain injury. J Neurol. 2019;266(10):2430–3.

[5] Arshad Q, Roberts RE, Ahmad H, Lobo R, Patel M, Ham T, et al. Patients with chronic dizziness following traumatic head injury typically have multiple diagnoses involving combined peripheral and central vestibular dysfunction. Clin Neurol Neurosurg. 2017;155:17–9.

[6] Yardley L, Beech S, Weinman J. Influence of beliefs about the consequences of dizziness on handicap in people with dizziness, and the effect of therapy on beliefs. J Psychosom Res. 2001;50(1):1–6.

[7] Kleffelgaard I, Langhammer B, Hellstrom T, Sandhaug M, Tamber AL, Soberg HL. Dizziness-related disability following mild–moderate traumatic brain injury. Brain Inj. 2017;31(11):1436–44.

[8] Killington MJ, Speck K, Kahlbaum J, Fabian J, Edwards D, Stobie J. Quality-of-life for individuals with a vestibular impairment following an acquired brain injury (ABI); the clients' perspective. Brain Inj. 2015 Mar 21;29(4):490–500.

[9] Chamelian L, Feinstein A. Outcome after mild to moderate traumatic brain injury: The role of dizziness. Arch Phys Med Rehabil. 2018 Jan 26;85(10):1662–6.

[10] McKechnie D, Pryor J, Fisher MJ. Falls and fallers in traumatic brain injury (TBI) rehabilitation settings: An integrative review. Disabil Rehabil. 2015;37(24):2291–9.

[11] McKechnie D, Pryor J, Fisher MJ. An examination of patient characteristics that

For peer review only

contribute to falls in the inpatient traumatic brain injury rehabilitation setting. Disabil Rehabil. 2017;39(18):1864–71.

[12] Agrawal Y, Carey JP, Della Santina CC, Schubert MC, Minor LB. Disorders of Balance and Vestibular Function in US Adults. Arch Intern Med. 2009;169(10):938.

[13] Murphy MP, Carmine H, Kolakowsky-Hayner S. Modifiable and Nonmodifiable Risk Factors for Falls After Traumatic Brain Injury: An Exploratory Investigation With Implications for Medication Use. Rehabil Nurs. 2014 May 1;39(3):113–22.

[14] Hartholt K, van Beeck E, MD P, Polinder S, van der Velde N, MD P, et al. Societal Consequences of Falls in the Older Population: Injuries, Healthcare Costs, and Long-Term Reduced Quality of Life. J Trauma. 2011;71(3):748–53.

[15] Gerber L, Quanhong N, Hartl R, Ghajar J. Impact of falls on early mortality from severe traumatic brain injury. J Trauma Manag Outcomes. 2009;3:9.

[16] Carlson K, Meis L, Jensen A, Simon A, Graveley A, Taylor B, et al. Caregiver reports of subsequent injuries among veterans with traumatic brain injury after discharge from inpatient polytrauma rehabilitation programs. J Head Trauma Rehabil. 2012;27(1):14–25.

[17] Indovina I, Riccelli R, Staab JP, Lacquaniti F, Passamonti L. Personality traits modulate subcortical and cortical vestibular and anxiety responses to sound-evoked otolithic receptor stimulation. J Psychosom Res. 2018 Jan 27;77(5):391–400.

[18] Indovina I, Riccelli R, Chiarella G, Petrolo C, Augimeri A, Giofrè L, et al. Role of the Insula and Vestibular System in Patients with Chronic Subjective Dizziness: An fMRI Study Using Sound-Evoked Vestibular Stimulation. Front Behav Neurosci. 2015 Dec 9;9:334.

[19] Riccelli R, Indovina I, Staab JP, Nigro S, Augimeri A, Lacquaniti F, et al. Neuroticism modulates brain visuo-vestibular and anxiety systems during a virtual rollercoaster

For peer review only

task. Hum Brain Mapp. 2017;38(2):715–26.

[20] Marroney N, Beattie J, Hildebrand N, Flint T, Smith RM. Does training therapists to manage benign paroxysmal positional vertigo in patients with acute traumatic brain injury reduce vestibular neurology referrals? Brain Inj. 2022 May;36(6):822–6.

[21] Hodgkinson S, Pollit V, Sharpin C, Lecky F. Early management of head injury: Summary of updated NICE guidance. BMJ. 2014;348(January):1–7.

[22] Calzolari E, Chepisheva M, Smith RM, Hellyer P, Tahtis V, Arshad Q, et al. Vestibular Agnosia in Traumatic Brain Injury and its Link to Imbalance. Brain. 2020;In Press.

[23] NHS England. NHS Standard Contract for Major Trauma Services. 2013.

[24] Rust HM, Smith RM, Mahmud M, Golding JF, Seemungal BM. Force dependency of benign paroxysmal positional vertigo in acute traumatic brain injury: a prospective study. J Neurol Neurosurg & Psychiatry. 2022 Apr 10;jnnp-2022-328997.

[25] Finch J. Robert Walker (ed.), Applied Qualitative Research, Gower, Aldershot, 1985. 203 pp. £17.50, paper £7.95. J Soc Policy. 2009/01/20. 1986;15(3):402–3.

[26] Creswell J. Qualitative Inquiry and Research Design: Choosing among five approaches. London: SAGE Publications; 2012. 40–66 p.

[27] Ritchie J, Lewis J. Qualitative Research Practice: A Guide for Social Science Students and Researchers. London: SAGE Publications; 2003.

[28] Gale NK, Heath G, Cameron E, Rashid S, Redwood S. Using the framework method for the analysis of qualitative data in multi-disciplinary health research. BMC Med Res Methodol. 2013;13(1):117.

[29] Sheard L, Prout H, Dowding D, Noble S, Watt I, Maraveyas A, et al. Barriers to the diagnosis and treatment of venous thromboembolism in advanced cancer patients: A qualitative study. Palliat Med. 2012 Oct 23;27(4):339–48.

For peer review only

[30] Marlow LA V, McGregor LM, Nazroo JY, Wardle J. Facilitators and barriers to help-seeking for breast and cervical cancer symptoms: a qualitative study with an ethnically diverse sample in London. Psychooncology. 2014 Jul 19;23(7):749–57.

[31] Marlow LA V, Waller J, Wardle J. Barriers to cervical cancer screening among ethnic minority women: a qualitative study. J Fam Plan Reprod Heal Care. 2015 Oct 12;41(4):248–54.

[32] Shaw D, Siriwardena AN. Identifying barriers and facilitators to ambulance service assessment and treatment of acute asthma: a focus group study. BMC Emerg Med. 2014 Aug 3;14:18.

[33] Kelleher E, Harrington JM, Shiely F, Perry IJ, McHugh SM. Barriers and facilitators to the implementation of a community-based, multidisciplinary, family-focused childhood weight management programme in Ireland: A qualitative study. BMJ Open. 2017;7(8):1–10.

[34] Homer CV, Tod AM, Thompson AR, Allmark P, Goyder E. Expectations and patients' experiences of obesity prior to bariatric surgery: A qualitative study. BMJ Open. 2016;6(2):1–10.

[35] Gale N, Sultan H. Telehealth as 'peace of mind': embodiment, emotions and the home as the primary health space for people with chronic obstructive pulmonary disorder. Health Place. 2013;21:140–7.

[36] Rashidian A, Eccles MP, Russell I. Falling on stony ground? A qualitative study of implementation of clinical guidelines' prescribing recommendations in primary care. Health Policy (New York). 2008;85(2):148–61.

[37] Regan T, Levesque J., Lambert S., Kelly B. A qualitative investigation of healthcare professionals', patients and partners views on psychosocial issues and related interventions for couples coping with cancer. PLoS One. 2015;10:1–22.

For peer review only

[38] Heath G, Cameron E, Cummins C, Greenfield S, Pattison H, Kelly D, et al. Paediatric 'care closer to home': Stake-holder views and barriers to implementation. Health Place. 2012;18(5):1068–73.

[39] Ayatollahi H, Bath PA, Goodacre S. Factors influencing the use of IT in the emergency department: A qualitative study. Health Informatics J. 2010 Sep 1;16(3):189–200.

[40] French SD, Green SE, O'Connor DA, McKenzie JE, Francis JJ, Michie S, et al. Developing theory-informed behaviour change interventions to implement evidence into practice: a systematic approach using the Theoretical Domains Framework. Implement Sci. 2012;7(1):38.

[41] Michie S, Johnston M, Abraham C, Lawton R, Parker D, Walker A. Making psychological theory useful for implementing evidence based practice: a consensus approach. Qual Saf Heal Care. 2005 Feb 1;14(1):26 LP – 33.

[42] Miles J, Gilbert P. A Handbook of Research Methods for Clinical & Health Psychology. Oxford: Oxford University Press; 2005.

[43] Shaw D, Siriwardena AN, Marlow LA V, McGregor LM, Nazroo JY, Wardle J, et al. Understanding diagnosis and management of dementia and guideline implementation in general practice: a qualitative study using the theoretical domains framework. Psychooncology. 2014 Jul 19;14(7):18.

[44] Decrop A. BIBLIOGRAPHIE: Qualitative Research Practice a Guide for Social Science Students and Researchers. Vol. 19, Recherche et Applications en Marketing (French Edition). London: SAGE Publications; 2004. 2–3 p.

[45] Tong A, Sainsbury P, Craig J. Consolidated criteria for reporting qualitative research (COREQ): a 32-item checklist for interviews and focus groups. Int J Qual Heal Care. 2007 Dec 1;19(6):349–57.

For peer review only

[46] Mañas MA, Díaz-Fúnez P, Pecino V, López-Liria R, Padilla D, Aguilar-Parra JM. Consequences of team job demands: Role ambiguity climate, affective engagement, and extra-role performance. Front Psychol. 2018;8(January):1–8.

[47] Hassan Z, Smith M, Littlewood S, Bouamra O, Hughes D, Biggin C, et al. Head injuries: a study evaluating the impact of the NICE head injury guidelines. Emerg Med J. 2005 Dec 1;22(12):845 LP – 849.

[48] Male A, Ramdharry G, Grant R, Davies RA, Beith ID. A survey of current management of Benign Paroxysmal Positional Vertigo (BPPV) by physiotherapists' interested in vestibular rehabilitation in the UK. Physiotherapy. 2018 Sep 1;105.

[49] Meldrum D, Burrows L, Cakrt O, Kerkeni H, Lopez C, Tjernstrom F, et al. Vestibular rehabilitation in Europe: a survey of clinical and research practice. J Neurol. 2020;

[50] Burrows L, Bryce K, Cole H, Haswell L, Metz D, Stevens K, et al. The ACPIVR Framework for Physiotherapists working within Vestibular and Balance System Health Care. 2021.

[51] Passi V, Johnson N. The impact of positive doctor role modeling. Med Teach. 2016 Nov 1;38(11):1139–45.

[52] Park J, Woodrow SI, Reznick RK, Beales J, MacRae HM. Observation, Reflection, and Reinforcement: Surgery Faculty Members' and Residents' Perceptions of How They Learned Professionalism. Acad Med. 2010;85(1).

[53] Polensek SH, Tusa RJ, Sterk CE. The challenges of managing vestibular disorders: a qualitative study of clinicians' experiences associated with low referral rates for vestibular rehabilitation. Int J Clin Pract. 2009 Nov 1;63(11):1604–12.

[54] Newman-Toker DE, Cannon LM, Stofferahn ME, Rothman RE, Hsieh Y-H, Zee DS. Imprecision in patient reports of dizziness symptom quality: a cross-sectional study conducted in an acute care setting. Mayo Clin Proc. 2007 Nov;82(11):1329–40.

For peer review only

[55] Olsson Möller U, Hansson EE, Ekdahl C, Midlöv P, Jakobsson U, Kristensson J. Fighting for control in an unpredictable life – a qualitative study of older persons' experiences of living with chronic dizziness. BMC Geriatr. 2014;14(1):97.

[56] Sezier AEI, Saywell N, Terry G, Taylor D, Kayes N. Working-age adults' perspectives on living with persistent postural-perceptual dizziness: a qualitative exploratory study. BMJ Open. 2019 Apr 1;9(4):e024326.

[57] Sharp DJ, Jenkins PO. Concussion is confusing us all. Pract Neurol. 2015 Jun 1;15(3):172 LP – 186.

[58] Herdman D, Evetovits A, Everton HD, Murdin L. Is "persistent postural perceptual dizziness" a helpful diagnostic label? A qualitative exploratory study. J Vestib Res. 2021;31(1):11–21.

[59] Petty RE, Briñol P, Tormala ZL. Thought confidence as a determinant of persuasion: the self-validation hypothesis. J Pers Soc Psychol. 2002 May;82(5):722—741.

[60] Harris L, Hateley S, Tsang KT, Wilson M, Seemungal BM. Impact of anti-epileptic drug choice on discharge in acute traumatic brain injury patients. J Neurol. 2020 Jun;267(6):1774–9.

[61] von Brevern M, Zeise D, Neuhauser H, Clarke AH, Lempert T. Acute migrainous vertigo: clinical and oculographic findings. Brain. 2005 Feb;128(Pt 2):365–74.

[62] J AD, G C. Healthcare professionals in research (HPiR) Facebook community: a survey of U.K. doctoral and postdoctoral healthcare professionals outside of medicine. BMC Med Educ. 2021;21(1):236.

[63] Cohen HS, Gottshall KR, Graziano M, Malmstrom E-M, Sharpe MH. International survey of vestibular rehabilitation therapists by the Barany Society Ad Hoc Committee on Vestibular Rehabilitation Therapy. J Vestib Res. 2009;19(1–2):15–20.

[64] Booth J, Hewison A. Role overlap between occupational therapy and physiotherapy

For peer review only

during in-patient stroke rehabilitation : an exploratory study Role overlap between occupational therapy and physiotherapy during in-patient stroke rehabilitation : an exploratory study. 2009;1820.

[65] Burrows L, Lesser TH, Kasbekar A V, Roland N, Billing M. Independent prescriber physiotherapist led balance clinic: the Southport and Ormskirk pathway. J Laryngol Otol. 2017/02/16. 2017;131(5):417–24.

[66] Jumani K, Powell J. Benign Paroxysmal Positional Vertigo: Management and Its Impact on Falls. Ann Otol Rhinol Laryngol. 2017 Aug;126(8):602–5.

[67] Ganança FF, Gazzola JM, Ganança CF, Caovilla HH, Ganança MM, Cruz OLM. Elderly falls associated with benign paroxysmal positional vertigo. Braz J Otorhinolaryngol. 2010;76(1):113–20.

[68] Davies P, Walker AE, Grimshaw JM. A systematic review of the use of theory in the design of guideline dissemination and implementation strategies and interpretation of the results in rigorous evaluations. Implement Sci. 2010;5:14–20.

[69] Van Bokhoven MA, Kok G, van der Weiden T. Designing a quality improvement intervention: A systematic approach. BMJ Qual Saf. 2003;12:215–20.

[70] Michie S, van Stralen MM, West R. The behaviour change wheel: A new method for characterising and designing behaviour change interventions. Implement Sci. 2011;6(1):42.

For peer review only

Figure 1. Five main themes relating to healthcare professionals' experiences of managing vestibular dysfunction and their relation to the overarching concept of role.

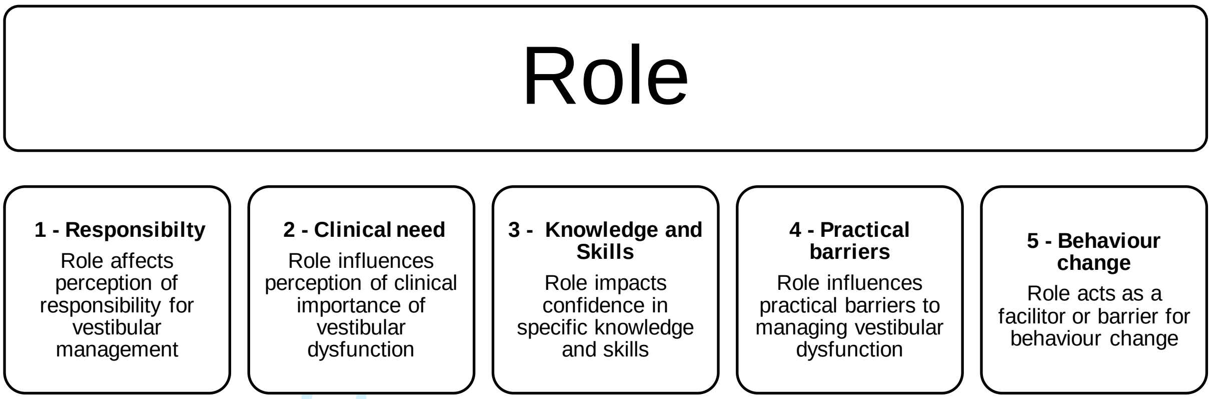

For peer review only

Figure 2. Diagram noting the themes contributing to the 'invisibility' of vestibular dysfunction

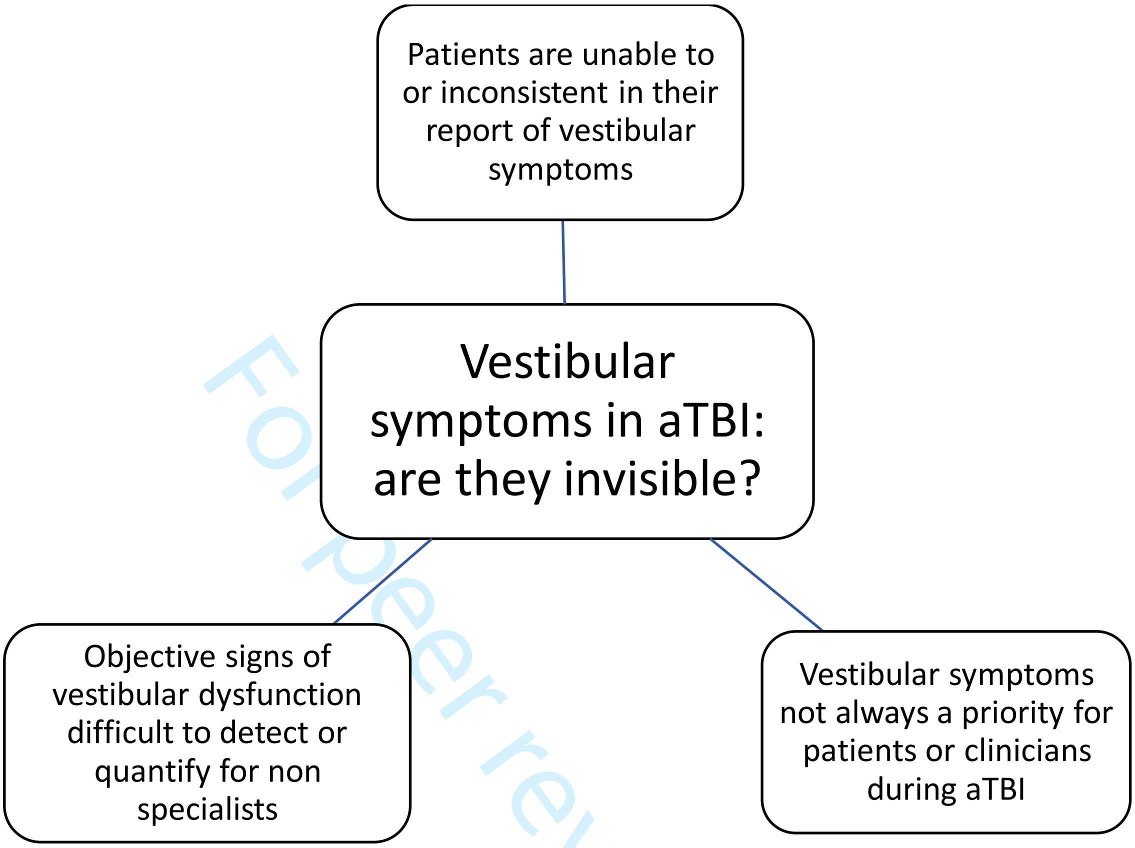

For peer review only

Supplementary Data

Supplementary File 1 - Topic guide

NHS Healthcare Professional Interview questions

Title: A qualitative study investigating the barriers and facilitators to screening of dizziness and imbalance in acute head injury patients.

Chief Investigator: Dr Barry Seemungal, Imperial College, Division of Brain Sciences, Charing Cross Hospital Campus, W6 8RF. Tel: 02033117042

Co-investigator: Rebecca Smith, Imperial College, Division of Brain Sciences, Charing Cross Hospital Campus, W6 8RF. Tel: 02033117042

<u>Introduction</u>

Thank you for agreeing to participate in this interview. The aim of the study is to explore healthcare professionals' knowledge and experience of assessing and treating dizziness in head injury patients. Your answers will be anonymous and will remain confidential. The interview will be audio recorded and I may take some notes whilst you are talking. Please keep in mind that you do not have to answer any questions you do not feel comfortable with, and we can stop the interview at any time.

<u>Background questions:</u>

1. Approximately how many patients with head injury with dizziness or imbalance do you see in a month? (i.e is dizziness a common symptom or sign after head injury?)

2. Can you talk me through the pathway of how head injury patients with dizziness or imbalance are managed?

3. Who is responsible for seeing these patients? (What do each of these team members do?)

4. What happens after they are assessed? (prompt: what is the treatment pathway? or onward referral procedure?)

We will now explore the pathway in detail using some more specific questions.

<u>Memory, attention and decision process</u>

5. Is assessing dizziness something you do routinely in head injury patients? (If not, why not?)

6. What factors might guide your decision to assess for dizziness in a head injured patient? (prompt: What clinical signs or symptoms are utilised?; formal assessments, on handover)

For peer review only

Knowledge

7. Which dizziness diagnoses are you familiar with post head injury? (Prompt: peripheral nerve injury; BPPV; migraine)

8. What sort of tests are you aware of that are useful in diagnosing dizziness? How are these test results used or interpreted? (prompt: are these standardized or validated tests?)

9. Are you aware of any treatment options for dizziness? (prompt: this might include onward referrals to other healthcare professionals)

10. Are there barriers to referring patients to appropriate services? (prompt: is there a wait for a specialist assessment?)

Skills

11. How confident are you in assessing and interpreting results of diagnostic tests for BPPV or other forms of dizziness?

12. What skills are required to treat dizziness conditions in head injury patients? (Prompt: BPPV)

Beliefs about capabilities

13. How would you rate your depth of understanding of dizziness? (prompt: How confident do you feel in managing dizziness after head injury?)

Social professional role and identity

14. Do you think conducting dizziness assessments is part of your role? If not, whose role does this fall into? (prompt: is assessing dizziness something you are trained to do?)

15. Do you think interpreting these results and providing treatment is an appropriate part of your role? Why / why not?

Beliefs about consequences

16. Are there any benefits of assessing and treating dizziness in these patients? (prompt: to yourself; patients, other clinicians; organisation).

17. Are there any consequences of 'not doing?' i.e. not assessing or treating? (prompt: is it a problem which resolves on its own; are there any consequences for the patient, clinicians, NHS, financial; long/short term) If no – why?

Motivation and goals

18. How important is it to assess and treat dizziness in head injury patients? (prompt: how high is the priority compared to other behaviours required to treat the patient?)

For peer review only

Environmental context and resources

19. Does the trauma ward situation or environment affect your management of dizzy head injury patients? (Prompt: i.e. are there time factors or competing tasks)

20. Are there clear communication channels if you are unsure of how to complete a test or interpret a result?

21. Are there any other person or environmental barriers or facilitators that might influence your screening behaviour? (prompt: guidelines/resources)

Social influences

22. Do other colleagues perform these tests? Does this influence your decision to complete / not complete these tests? (Prompt: AHPs; other medics)

23. How might the views of your colleagues affect you managing a dizzy head injured patient?

Emotion

24. Are there any situations in which you would be worried about managing dizziness in this population? (i.e medically/professionally/emotionally)

25. Are there any challenges in managing a head injury patient with dizziness?

Behavioral regulation

26. If you wanted to change your dizziness assessment behavior, how would you go about doing this? (prompt: what would facilitate you to do this?)

27. Is there any training that you or your team may require? (prompt: any procedures/guidelines/ways of working)

Lastly, is there any further you would like to add which we have not covered?

Thank you for participating in this interview.

For peer review only

Supplementary File 2 - Framework for analysis

1 Current practice

1.1 Identification and reporting of dizziness
1.2 Assessment and the assessment pathway
1.3 Treatment and referral pathway
1.4 Longer term effects of treating/not treating
1.5 Perceptions regarding role and responsibility relating to dizziness assessment and treatment
1.6 Views about specialist services

2 Dizziness and the clinical need

2.1 Factors relating to dizziness frequency, severity and longevity
2.2 Perceptions around the importance and clinical significance of dizziness
2.3 Impact of dizziness on patients
2.4 Impact of dizziness on service
2.5 Factors relating to early identification and intervention
2.6 Views about specific populations i.e. elderly or young

3 Current knowledge

3.1 Knowledge about BPPV as a condition
3.2 Knowledge and clinical reasoning relating to other causes of dizziness
3.3 Knowledge about diagnosis and treatment of BPPV
3.4 Healthcare professional’s ability to diagnose and treat dizziness conditions
3.5 Efficacy of BPPV treatment

4 Barriers to diagnosis and treatment competence

For peer review only

4.1 Views about the area and motivation/interest in the area
4.2 Factors related to training, skills or knowledge
4.3 Feasibility of diagnosis and treatment
4.4 Confidence
4.5 Role concerns
4.6 Prior surgical / medical clearance
4.7 Environmental factors
4.8 Lack of access to mentors or specialists
4.9 Tangible evidence of dizziness
4.10 Views about resources – staffing, workload, time

5 Changing behaviour and practice

5.1 Factors relating to role change
5.2 Facilitators to behaviour change
5.3 Benefits of practice change
5.4 Limitations or barriers to practice change
5.5 Training requirements
5.6 Strategies for changing or improving practice
5.7 Wider translatable aspects of behaviour change

For peer review only

Supplementary data 3 – Central Chart

	Profession	Gender	Speciality	Current role	View on clinical need	Assessment/Treatment knowledge & ability	Main barrier	Readiness for change
1 : AM12_1205	Medic, Junior Doctor	Male	Trauma	Adhoc, unspecific assessor, referrer	Low-medium	Partly theoretical, non functional	Resources & Clinical need	Reservations, not feasible to take on role
2 : AM13_1635	Medic, Registrar	Male	Trauma	Inactive, reliance on therapists	Low-medium	Theoretical, partly functional	Resources & Clinical need (view of the area)	Reservations, not feasible to take on role
3 : AM14_1530	Medic, Junior Doctor	Male	Trauma	Inactive	Low-medium	Partly theoretical, non functional	Current role & remit Resources & Clinical need	Reservations, not feasible to take on role (or just not engaged?)
4 : AM16_1445	Medic, Registrar	Female	Trauma	Inactive; reliance on therapists	Low-medium	Non theoretical, non functional	Resources & clinical need	Reservations, not feasible to take on role
5 : AM17_1700	Medic, Junior Doctor	Male	Trauma	Adhoc unspecific assessor, referrer	Low-medium	Non theoretical, non functional	Current role & remit	Reservations, not feasible to take on role
6 : AM21_1500	Medic, Junior doctor	Female	Trauma	Referrer	Low-medium	Non theoretical, non functional	Resources & clinical need	Minor reservations, not feasible to take on role
7 : AM271500	Medic, Registrar	Male	Trauma	Adhoc, unspecific assessor, referrer	Low-medium	Partly theoretical, non functional	Current role & remit	Reservations, not feasible to take on role
8 : AM281555	Medic, Junior Doctor	Female	Trauma	Adhoc, unspecific assessor, referrer	Low-medium	Partly theoretical, non functional	Resources & Clinical need	Reservations, not feasible to take on role
9 : AM8_1430	Medic, Consultant	Male	Trauma	Subjective/Objective identifier, unspecific assessor, referrer	Low-medium	Partly theoretical, non functional	Knowledge and skills	Reservations, not feasible to take on role
10 : AO1_1535	OT, Senior	Female	Trauma	Subjective/Objective identifier, unspecific assessor and referrer	High	Partly theoretical, non functional	Knowledge and skills	No reservations, keen to take on role
11 : AO15_1600	OT, Junior	Male	A&E	Routine identifier, unspecific assessor, referrer	Medium-high	Partly theoretical, non functional	Current role & remit	Reservations, not feasible to take on role
12 : AO18_1520	OT, Senior	Female	A&E	Routine identifier, unspecific assessor, referrer	Medium-high	Partly theoretical, non functional	Current role & remit	Reservations, not feasible to take on role
13 : AO19_1200	OT, Senior	Female	Neurology	Subjective identifier, unspecific assessor, referrer	Medium-high	Non theoretical, non functional	Current role & remit	Some reservations, uncertain about taking on the role
14 : AO20_1330	OT, Senior	Female	A&E	Subjective identifier, unspecific assessor, referrer	High	Theoretical, non functional	Current role & remit	Some reservations, uncertain about taking on role
15 : AO221447	OT, Senior	Female	Trauma	Routine identifier, screen, referrer	Medium-high	Theoretical, non functional	Current role & remit Confidence	Minor reservations, keen to take on role
16 : AO241030	OT, Senior	Female	Critical care	Subjective identifier, unspecific assessor, referrer	Low-medium	Theoretical, non functional	Current role & remit	Minor reservations, keen to take on role

For peer review only

17 : AO251130	OT, Senior	Male	Critical care	Subjective / objective identifier, unspecific assessor, referrer	Low-medium	Partly theoretical, non functional	Current role & remit	Minor reservations, keen to take on role
18 : AO4_1210	OT, Senior	Female	Trauma	Routine identifier, unspecific assessor, referrer	Medium-high	Partly theoretical, non functional	Current role & remit Knowledge and skills	Some reservations, uncertain about taking on role
19 : AO6_1005	OT, Senior	Female	Trauma	Routine identifier, unspecific assessor	High	Theoretical, non functional	Current role and remit Knowledge and skills	Minor reservations, keen to take on role.
20 : AO9_1230	OT, Junior	Female	Neurology	Routine identifier, unspecific assessor, referrer	Medium-high	Partly theoretical, non functional	Current role & remit	Minor reservations, keen to take on role
21 : AP10_2401	PT, Junior	Female	Neurology	Subjective identifier, unspecific assessor	Medium-high	Partly theoretical, non functional	Current role and remit Knowledge and skills	Some reservations, uncertain about taking on role
22 : AP11_1115	PT, Senior	Female	Trauma	Routine identifier, unspecific assessor, referrer	High	Theoretical, non functional	Low confidence	Minor reservations, keen to take on role
23 : AP2_1541	PT, Junior	Female	Neurology	Subjective identifier, unspecific assessor, referrer	Medium	Partly theoretical, non functional	Knowledge and skills	Minor reservations, keen to take on role
24 : AP231544	PT, Senior	Female	Neurology	Routine identifier, specific assessor, treat	Medium	Theoretical and partly functional	Confidence	Minor reservations, keen to take on role
25 : AP261400	PT, Senior	Female	Trauma	Subjective/objective identifier, unspecific assessor, referrer	Low-Medium	Theoretical, non functional	Current role & remit	Minor reservations, keen to take on role
26 : AP3_1030	PT, Junior	Male	Trauma	Subjective/objective identifier, referrer	High	Theoretical, partly functional	Confidence	Minor reservations, keen to take on role
27 : AP5_0840	PT, Junior	Female	Trauma	Subjective/objective identifier, unspecific assessor, referrer	Medium-high	Partly theoretical, non functional	Knowledge and skills	Minor reservations, keen to take on role
28 : AP7_1515	PT, Senior	Female	Trauma	Routine identifier, semi specific assessor, referrer	Medium-high	Partly theoretical, non functional	Knowledge and skills	Minor reservations, keen to take on role

Key: OT (Occupational Therapist); PT (Physiotherapist); Subjective/objective/routine identifier (referring to healthcare professional who is involved in routinely identifying patients with potential vestibular dysfunction through either subjective or objective means); specific/unspecific assessor (referring to a healthcare professional able to assess vestibular dysfunction using specific, semi specific or non specific assessment tools); referrer (referring to healthcare professional who refers onto specialists for further management); Theoretical (referring to healthcare professionals with theoretical knowledge of vestibular diagnoses, assessment and treatment); Functional /non functional (referring to healthcare professionals who utilise their vestibular knowledge and skills to assess and treat patients)

For peer review only

For peer review only

For peer review only

COREQ (COnsolidated criteria for REporting Qualitative research) Checklist

A checklist of items that should be included in reports of qualitative research. You must report the page number in your manuscript where you consider each of the items listed in this checklist. If you have not included this information, either revise your manuscript accordingly before submitting or note N/A.

Topic	Item No.	Guide Questions/Description	Reported on Page No.
**Domain 1: Research team and reflexivity**			
Personal characteristics			
Interviewer/facilitator	1	Which author/s conducted the interview or focus group?	
Credentials	2	What were the researcher's credentials? E.g. PhD, MD	
Occupation	3	What was their occupation at the time of the study?	
Gender	4	Was the researcher male or female?	
Experience and training	5	What experience or training did the researcher have?	
Relationship with participants			
Relationship established	6	Was a relationship established prior to study commencement?	
Participant knowledge of the interviewer	7	What did the participants know about the researcher? e.g. personal goals, reasons for doing the research	
Interviewer characteristics	8	What characteristics were reported about the inter viewer/facilitator? e.g. Bias, assumptions, reasons and interests in the research topic	
**Domain 2: Study design**			
Theoretical framework			
Methodological orientation and Theory	9	What methodological orientation was stated to underpin the study? e.g. grounded theory, discourse analysis, ethnography, phenomenology, content analysis	
Participant selection			
Sampling	10	How were participants selected? e.g. purposive, convenience, consecutive, snowball	
Method of approach	11	How were participants approached? e.g. face-to-face, telephone, mail, email	
Sample size	12	How many participants were in the study?	
Non-participation	13	How many people refused to participate or dropped out? Reasons?	
Setting			
Setting of data collection	14	Where was the data collected? e.g. home, clinic, workplace	
Presence of non-participants	15	Was anyone else present besides the participants and researchers?	
Description of sample	16	What are the important characteristics of the sample? e.g. demographic data, date	
Data collection			
Interview guide	17	Were questions, prompts, guides provided by the authors? Was it pilot tested?	
Repeat interviews	18	Were repeat inter views carried out? If yes, how many?	
Audio/visual recording	19	Did the research use audio or visual recording to collect the data?	
Field notes	20	Were field notes made during and/or after the inter view or focus group?	
Duration	21	What was the duration of the inter views or focus group?	
Data saturation	22	Was data saturation discussed?	
Transcripts returned	23	Were transcripts returned to participants for comment and/or	

For peer review only

Topic	Item No.	Guide Questions/Description	Reported on Page No.
		correction?	
**Domain 3: analysis and findings**			
Data analysis			
Number of data coders	24	How many data coders coded the data?	
Description of the coding tree	25	Did authors provide a description of the coding tree?	
Derivation of themes	26	Were themes identified in advance or derived from the data?	
Software	27	What software, if applicable, was used to manage the data?	
Participant checking	28	Did participants provide feedback on the findings?	
Reporting			
Quotations presented	29	Were participant quotations presented to illustrate the themes/findings? Was each quotation identified? e.g. participant number	
Data and findings consistent	30	Was there consistency between the data presented and the findings?	
Clarity of major themes	31	Were major themes clearly presented in the findings?	
Clarity of minor themes	32	Is there a description of diverse cases or discussion of minor themes?	

Developed from: Tong A, Sainsbury P, Craig J. Consolidated criteria for reporting qualitative research (COREQ): a 32-item checklist for interviews and focus groups. *International Journal for Quality in Health Care*. 2007. Volume 19, Number 6: pp. 349 – 357

Once you have completed this checklist, please save a copy and upload it as part of your submission. DO NOT include this checklist as part of the main manuscript document. It must be uploaded as a separate file.

For peer review only
